# Small-Occupation Density Functional Correlation Energy Correction to Wave Function Approximations

**DOI:** 10.1021/acs.jctc.3c01067

**Published:** 2024-01-16

**Authors:** José Aarón Rodríguez-Jiménez, Abel Carreras, David Casanova

**Affiliations:** † 226245 Donostia International Physics Center (DIPC), 20018 Donostia, Euskadi, Spain; ‡ Multiverse Computing, 20008 Donostia, Euskadi, Spain; § Polimero eta Material Aurreratuak: Fisika, Kimika eta Teknologia, Kimika Fakultatea, Euskal Herriko Unibertsitatea (UPV/EHU), 20018 Donostia, Euskadi, Spain; ∥ IKERBASQUE, Basque Foundation for Science, 48009 Bilbao, Euskadi, Spain

## Abstract

In this work, we introduce a novel hybrid approach, termed WFT-*so*DFT, designed to seamlessly incorporate DFT correlation into wave function ansatzes. This is achieved through a partitioning of the orbital space, distinguishing between large and small natural occupation numbers associated with wave function theory (WFT) and DFT correlation, respectively. The method uses a novel criterion for partitioning the orbital space and mapping the electron density in natural orbitals with a small occupation with the correlation energy of fast electrons within the homogeneous electron gas. Central to our approach is the introduction of a separation parameter ν, the choice of the WFT approach, and the correlation functional. Here, we combine the RASCI wave function with *hole* and *particle* truncation with a local density correlation functional to only account for small-occupation correlation energy. We investigate the performance of the method in the study of small but challenging chemical systems, for which WFT-*so*DFT demonstrates notable improvements over pristine wave function calculations. These findings collectively highlight the potential of the WFT-*so*DFT approach as a computationally affordable strategy to improve the accuracy of WFT electronic structure calculations.

## Graphical abstract

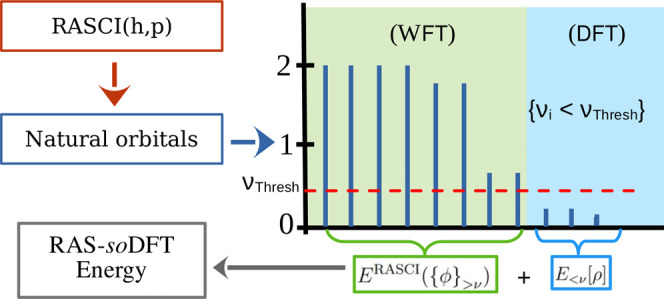

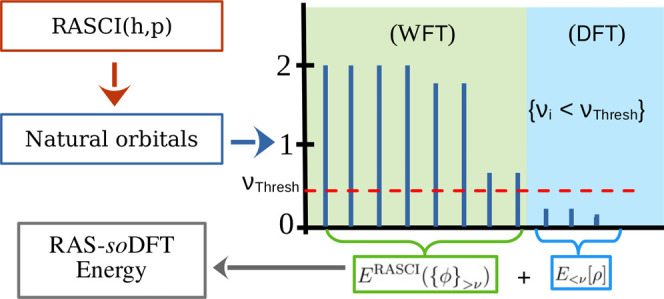

## 1 Introduction

Molecular electronic structure descriptions traditionally rely on the nonrelativistic framework and the Born–Oppenheimer approximation, simplifying the treatment of nuclei and electrons to solving the electronic time-independent Schrödinger equation. Although the full configuration interaction (FCI) offers an exact solution (in a given one-electron basis), its computational demands limit its applicability to small systems.^1–3^ These limitations have spurred the development of approximate quantum chemistry methods, with a focus on reducing the dimensionality of the problem in two main directions: (i) employing finite basis sets for the description of single electrons, which already implies a level of approximation with respect to the exact solution^4^ and (ii) addressing electron–electron interactions, also known as the electron correlation problem.^5^ Among the latter, Hartree–Fock (HF) theory provides a qualitative solution for electronic ground states,^6^ capturing a large portion of the total electronic energy in closed-shell molecules. Post-HF methods build on this foundation, employing perturbation theory (e.g., MP2), configuration interaction (CI), or coupled cluster (CC) methods [as in CCSD^7^ or CCSD­(T)^8^]. However, HF theory struggles in strongly correlated systems, necessitating multiconfigurational approaches like the complete active-space self-consistent field (CASSCF)^9^ or its perturbative variant (CASPT2),^10^ which are computationally intensive and thus restricted to smaller molecules. On the other hand, density functional theory (DFT), in particular in the Kohn–Sham scheme,^11^ offers an efficient alternative by bypassing the need for many-body wave functions. However, the lack of an exact universal energy functional limits the accuracy of DFT approximations,^12^ especially in cases of degeneracies or near degeneracies.

Given the distinct capabilities of wave function theory (WFT) and DFT in capturing static and dynamic correlations, respectively, there has been interest in combining these approaches.^13^ Common mingled WFT-DFT models^14^ rely on range separation of the Coulomb operator,^15,16^ on the multiconfiguration pair-density functional theory (MC-PDFT),^17,18^ or on the multiconfiguration density coherence functional theory (MC-DCFT),^19^ showing promise in various chemical scenarios. Alternatively, it has been shown that the eigenstates of the density matrix, i.e., natural orbitals (NOs), and their occupation numbers (NOONs) can be used to characterize correlation effects.^20^ Analysis of NOONs has inspired novel strategies for active-space selection,^21–24^ and the distribution of NOONs has been used to characterize strongly correlated systems^25^ and as a metric to quantify the open-shell character of electronic wave functions.^26–29^ Results from condensed matter physics, where strongly correlated electrons are usually described with model Hamiltonians,^30^ and the homogeneous electron gas (HEG), where the NOs are plane waves,^20^ show that large NOONs describe molecule-specific effects, while those orbitals with small occupations and large momentum can be related to short-range effects. These properties were exploited by Savin, who designed a strategy to mix WFT and DFT by splitting the orbital space between those NOs with NOONs larger than a given threshold (evaluated with WFT) and those with small NOONs (used to quantify electron correlation with DFT).^31–34^ However, since those initial works, no further investigations have been carried out in this direction. In the present work, we reformulate the correction of multiconfigurational wave functions with DFT correlation through orbital space separation. We aim to establish a general formalism to accommodate suitable WFT models and DFT functionals based on the splitting of the orbital space, termed WFT with small-occupation DFT (WFT-*so*DFT), and evaluate its performance, addressing its advantages and limitations in characterizing chemical systems.

The article is organized as follows: First, we introduce and describe the theoretical framework for the mingling of WFT with DFT (Section 2), we motivate the partition of the orbital space as a justified strategy, and we discuss several aspects of the methodology (Section 3). Section 4 describes the workflow of the WFT-*so*DFT method and the employed computational details and nomenclature. Section 5 addresses the performance of the computation of the He and Be atomic series, the single bond breaking in H_2_ and C_2_H_4_, and multiple bond dissociation in N_2_ dissociation. The main findings of the work are summarized in the Conclusions section.

## 2 General Framework to Merge WFT with DFT

In WFT, the exact (nonrelativistic) ground-state energy for an electronic molecular system can be obtained variationally as

1 $$E_{0}\equiv E_{0}\left[\Psi_{0}\right]=\underset{\Psi }{\min}\left\{\langle \Psi \left|\mathrm{T̂}+{\mathrm{V̂}}_{ne}+{\mathrm{V̂}}_{ee}\right|\Psi \rangle \right\}$$

where Ψ_0_ is the exact ground-state wave function, *T̂* is the kinetic energy operator, ${\mathrm{V̂}}_{ne}$ and ${\mathrm{V̂}}_{ee}$ are the operators, respectively, accounting for nuclei–electron and electron–electron interactions, and the minimization runs over all possible antisymmetric wave functions, i.e., the entire Hilbert space. In practice, the minimization in eq 1 is conducted within a finite Hilbert subspace (*S*) defined by the electron correlation approximation and the chosen basis set

$$E_{0}^{WFA}=\underset{\mathrm{\Psi ̃}\in S}{\min}\left\{\langle \mathrm{\Psi ̃}\left|\mathrm{T̂}+{\mathrm{V̂}}_{ne}+{\mathrm{V̂}}_{ee}\right|\mathrm{\Psi ̃}\rangle \right\}$$ 2

The fairness of the wave function approximation (WFA) is given by the difference between the exact and approximate energies. In particular, for mean-field wave functions, i.e., HF, the difference with respect to the exact energy is called *electron correlation energy*. More concretely, for wave functions capturing the main electronic configurations, e.g., HF (only one) or CASSCF^9^ with small or moderate active spaces, the energy error of the approximate wave function is labeled as *dynamic correlation energy*.^35,36^

Alternatively, the Hohenberg–Kohn theorem^37^ allows the expression of the ground-state energy as a function of the electron density

3 $$E_{0}\left[\rho \right]=F_{0}\left[\rho \right]+\int v\left(r\right)\rho \left(r\right)\;dr$$

where *v*(*r*) is the external potential, $v\left(r\right)=\left\langle \Psi \left|{\mathrm{V̂}}_{ne}\right|\Psi \right\rangle$ , and *F*_0_[ρ] is the universal Levy–Lieb (LL) density functional^38,39^

4 $$F_{0}\left[\rho \right]=\min_{\Psi \to \rho }\left\{\langle \Psi \left|\mathrm{T̂}+{\mathrm{V̂}}_{ee}\right|\Psi \rangle \right\}$$

with the minimization performed over all wave functions with density ρ. Then, the exact ground-state energy can be obtained by minimizing the energy functional in eq 3

5 $$E_{0}\equiv E_{0}\left[\rho_{0}\right]=\min_{\rho }\left\{F_{0}\left[\rho \right]+\int v\left(r\right)\rho \left(r\right)\;dr\right\}$$

where ρ_0_ is the exact ground-state density, ρ_0_ ≡ ρ­[Ψ_0_]. If the LL functional is obtained only considering a restricted wave function space, Ψ̃ ∈ *S*, and assuming that the restricted space *S* contains at least one wave function Ψ̃ able to generate ρ

6 $${\mathrm{F̃}}_{0}\left[\rho \right]=\min_{\mathrm{\Psi ̃}\to \rho }\left\{\langle \mathrm{\Psi ̃}\left|\mathrm{T̂}+{\mathrm{V̂}}_{ee}\right|\mathrm{\Psi ̃}\rangle \right\}$$

then, it is possible to define a correlation energy functional, *E*_c_[ρ], accounting for the missing energy to the exact ground state

7 $$E_{c}\left[\rho \right]=E_{0}\left[\rho \right]-{Ẽ}_{0}\left[\rho \right]=F_{0}\left[\rho \right]-{\mathrm{F̃}}_{0}\left[\rho \right]$$

Hence, the exact ground-state energy can be formally obtained by computing the approximate and correlation functionals at ρ_0_. In practice, however, one does not have access to ρ_0_. Therefore, we approximate the exact energy by evaluating each term at the approximate density, that is, the optimal density within the restricted space, ${\mathrm{\rho ̃}}_{0}\equiv \rho \left[{\mathrm{\Psi ̃}}_{0}\right]$

8 $$E_{0}\approx {Ẽ}_{0}\left[{\mathrm{\rho ̃}}_{0}\right]+E_{c}\left[{\mathrm{\rho ̃}}_{0}\right]$$

Equation 8 can be used to define new methods mixing approximate wave functions with DFT by assigning the calculation of the approximate ground-state energy to WFA (eq 2)­

9 $$E_{0}≃E_{0}^{WFA}+E_{c}\left[{\mathrm{\rho ̃}}_{0}\right]$$

In general, for a good enough trial wave function ${\mathrm{\Psi ̃}}_{0}$ , one should expect the errors derived from the explicit form of *E*_c_[ρ] to be larger than those derived by the use of approximate densities, thus justifying the use of eq 8.

We must stress that this strategy relies on the proper separation of the WFT and DFT terms in order to compute complementary contributions to the electronic energy while avoiding double counting of correlation effects. In the present work, we explore the use of the orbital space to split the total electronic energy in WFT and DFT contributions.

## 3 Orbital Separation Based on Electron Occupation

Equation 9 provides a general framework for the mixing of WFT and DFT. However, it is nowhere close to being a working equation. Reliable choices for explicit forms of the two contributions in the RHS of eq 9, i.e., approaching the exact energy $\left(E_{0}\right)$ , should consider the properties and limitations of the available WFAs and DFAs. Hence, we envision the use of WFA to account for strong (nondynamic) correlation effects (difficult to recover with energy density functionals),^40,41^ leaving dynamic correlation to DFA, for which WFAs typically show slow convergence.

The direct additive combination of WFA energy with the correlation energy from DFT, although tempting, has been shown to be inappropriate, since the DFT correlation contribution might include correlation effects already present in the wave function part and vice versa (double counting of electron correlations).^42^ Therefore, it is necessary to employ schemes for the proper separation of the two contributions. To that end, we rely on the momentum distribution [*n*(*k*)] of the HEG, with a general profile shown in Figure 1, corresponding to a distribution of the electron occupation expressed in terms of the NOs of the system (plane waves). The probability of finding an electron with *k* < *k*_F_ is close to one up until the electron momentum approaches the Fermi wavevector (*k*_F_), where it exhibits a small decay with a profile depending on the electron density,^43^ as characterized by the Wigner–Seitz radius (*r*_s_). The electron density of the HEG presents a discontinuity at *k*_F_. For the case of interacting electrons, the momentum distribution is *n* ^+^ ≡ *n*(*k* → *k*_F_^+^, *r*_s_) ≠ 0 (+ superscript indicates right-handed limit), whereas *n*(*k*) rapidly decays as *k* → ∞.

**Figure 1:**
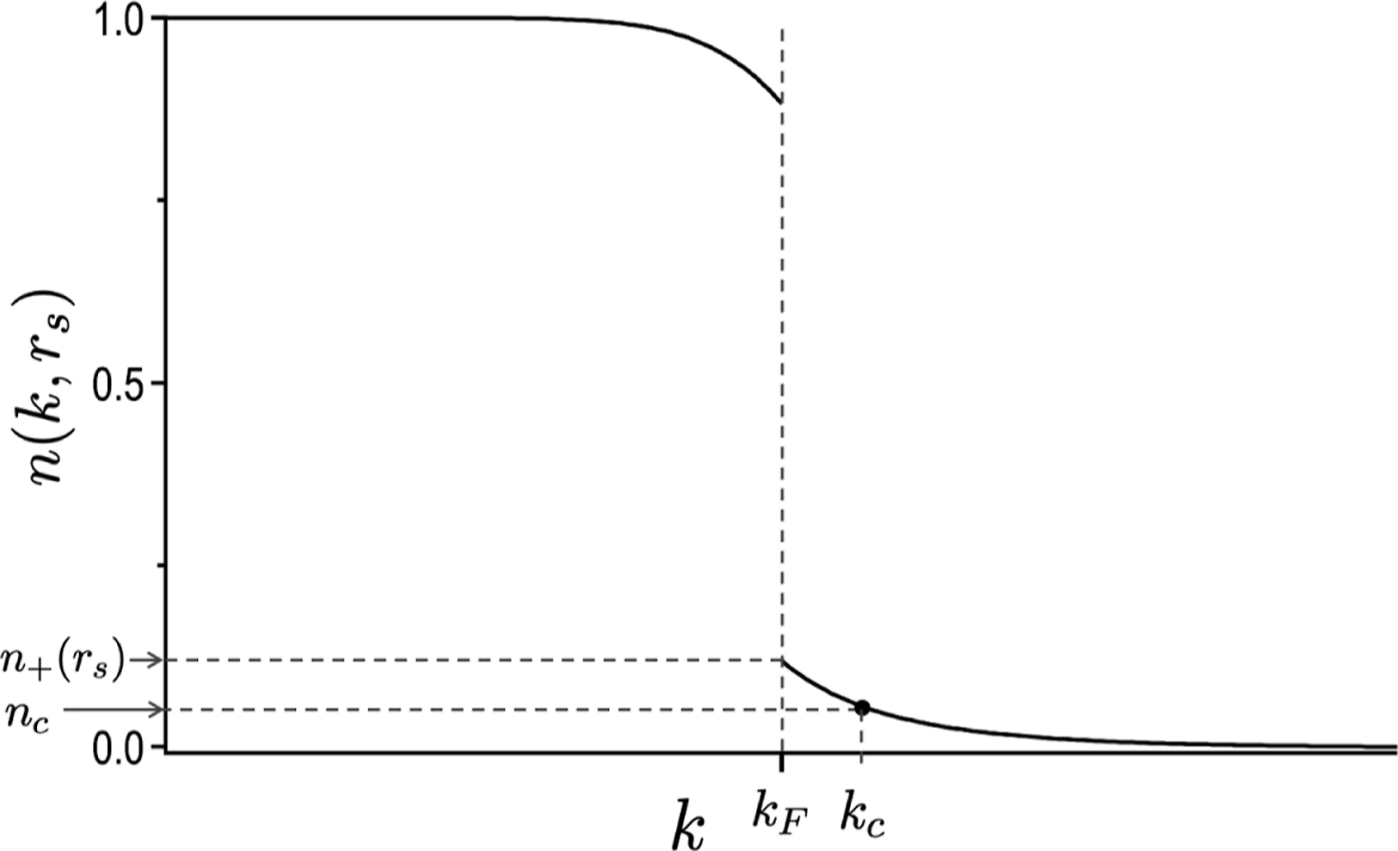
Representation of the momentum distribution for the HEG. Two points are highlighted, the first at the Fermi wavevector (*k*_F_) with its associated probability limit approaching from *k* > *k*_F_ [*n*_+_(*r*_s_) = *n*(*k*_F_^+^, *r*_s_)] and the second with momentum *k*_C_ > *k*_F_ and probability *n*_C_ that corresponds to the point from which the dynamic correlation can be accounted for by the DF.

It has been argued that the dynamic correlation in the HEG can be associated with the high kinetic energy electrons, that is, to those NOs with small occupancies.^20^ This idea suggests a NO-based criterion to split between nondynamic and dynamic correlations, which can be, respectively, addressed by WFA and DFA. Savin connected such a behavior of the HEG to the study of atomic and molecular systems by suggesting to compute the contribution to the electron energy of NOs with high NOONs with a WFA able to recover the system-specific electron correlation effects and evaluate the (dynamic) correlation energy of NOs with small NOONs with DFT correlation energy functionals.^31,32^ Accordingly, it is possible to define a WFT–DFT composite approach, that we call *small-occupation DFT corrected WFT* (WFT-*so*DFT), by applying the occupation-based orbital splitting to eq 9

10 $$E^{WFT‐soDFT}=E_{0}^{WFA,\nu }+E_{c}^{so,\nu }\left[\rho \right]$$

11 $$E_{0}^{WFA,\nu }=\min_{\Psi^{\nu }}\left\langle \Psi^{\nu }\left|\mathrm{T̂}+{\mathrm{V̂}}_{ne}+{\mathrm{V̂}}_{ee}\right|\Psi^{\nu }\right\rangle$$

where ν is the (positive-definite) NOON separation parameter, i.e., the molecular counterpart of *n*_C_ for the HEG in Figure 1, and Ψ^ν^ is the wave function obtained by the chosen WFA but only considering NOs with NOONs > ν. The superindex ν explicitly indicates the dependence of the wave function and the correlation functional on the ν parameter. Three main issues need to be considered in the use of eq 10: (i) the choice of the occupation separation parameter ν; (ii) the design of correlation energy functionals (only) corresponding to NOs with low NOONs; and (iii) the accurate description of the NOONs’ distribution of the system. These three key aspects of WFT-*so*DFT are discussed in the following subsections.

### 3.1 Occupation Separation Parameter

The choice of the occupation threshold ν splitting the orbital space in WFT-*so*DFT is controlled by the ν parameter. It is important to notice that eq 10 represents a correction to the WFA. In the limit of ν → 0, the method converges to the WFT, while the ν → ∞ limit provides the total DFT electron correlation energy.

In the original formulation,^31,32^ the choice of ν was related to the idea of the existence of a universal threshold value ν_c_, which in practice can be used to define the system-dependent ν as

12 $$\nu =\min_{n_{i}}\left\{n_{i}\leq \nu_{c}\right\}$$

Numerical analysis on neutral systems recommended the use of ν_c_ ≈ 0.01 a.u.^32^ Such an approach, although simple, only considers the NOON occupation of a single NO and completely disregards the rest of the small-occupation NOs. To overcome these potential limitations, we introduce a new definition of ν by considering the entire tail of small NOONs. For that, we define ν through eq 13

13 $$\int_{k_{c}}^{\infty }n\left(k,r_{s}\right)\;dk=\frac{1}{N}\overset{n_{i}\leq \nu_{c}}{\underset{i}{\sum }}n_{i}$$

where

14 $$\int_{0}^{\infty }n\left(k,r_{s}\right)\;dk=1$$

*k* is in units of *k*_F_, {*n*_*i*_} are the NOONs obtained from the diagonalization of the one-particle density matrix (1-PDM), and *N* is the total number of electrons. Compared to eq 12, we envision eq 13 to be more robust to the specificities of the discrete distribution of NOONs as obtained with WFAs, e.g., NOONs’ degeneracies around ν_c_, and that it might represent a more balanced manner to include the *so*DFT correlation energy. Then, ν can be obtained either as ν = *n*(*k*_c_, *r*_s_) (defining the ν_c_ threshold and computing *k*_c_ with eq 13) or alternatively as ν = ν_c_ (defining *k*_c_ and computing ν_c_ from eq 13).^44^

The use of eq 13 requires an explicit expression for *n*(*k*, *r*_s_) distribution within the *k*_c_ ≤ *k* ≤ ∞ range. Gori-Giorgi and Ziesche^45^ have shown that *n*(*k*, *r*_s_) for *k* > *k*_F_ can be approximated as

15 $$n\left(k>k_{F},r_{s}\right)\approx n_{+}\left(r_{s}\right)\frac{n\left(k\to \infty ,r_{s}\right)}{n\left(k\to \infty ,r_{s}\right){|}_{k=k_{F}}}$$

where *n*_+_(*r*_s_) = *n*(*k*_F_^+^, *r*_s_) (Figure 1). The momentum distributions at the *k* → ∞ limit are known to decay proportionally to *k* ^–8^ ^46^ ,^47^

16 $$n\left(k\to \infty ,r_{s}\right)=\frac{8{\left(\alpha_{0}r_{s}\right)}^{2}g_{0}\left(r_{s}\right)}{9\pi k^{8}}+O\left(\frac{1}{k^{10}}\right)$$

where α_0_ is the Bohr radius and *g*_0_(*r*_s_) is the value of the pair-distribution function at zero-interelectronic distance, which can be parametrized as^48^

17 $$g_{0}\left(r_{s}\right)=\frac{1-Br_{s}+Cr_{s}^{2}+Dr_{s}^{3}+Er_{s}^{4}}{2}\;e^{-dr_{s}}$$

with parameters *d* = 0.7524, *B* = 0.7317 – *d*, *C* = 0.08193, *D* = −0.01277, and *E* = 0.001859. At this point, only a suitable expression for *n*_+_(*r*_s_) remains to be defined. Here, we will consider the form proposed by Savin^31^

18 $$n_{+}\left(r_{s}\right)=\frac{r_{s}}{r_{s}+8.45}$$

and the one given by Gori-Giorgi and Ziesche^45^

19 $$n_{+}\left(r_{s}\right)=\frac{q_{1}r_{s}}{1+q_{2}r_{s}^{1/2}+q_{3}r_{s}^{7/4}}$$

where *q*_1_ = 0.088519 (from RPA), *q*_2_ = 0.45, and *q*_3_ = 0.022786335.

It is worth noting that the orbital separation strategy discussed here might not preserve additive separability. Evaluation of size consistency errors in the WFT-*so*DFT scheme can be found in the Supporting Information.

### 3.2 Small-Occupation Correlation Energy Functionals

The challenging aspect in the design of small-occupation correlation energy functionals (*E*_c_^*so*,ν^[ρ]) is related to the need to exclude the contribution of NOs with large NOONs from the DFT correlation. To that end, Savin proposed a ν-dependent correlation functional that within the local density approximation (LDA) takes the form

$$E_{c}^{so,\nu }\left[\rho \right]=\int \rho \varepsilon_{c}\left(\rho \right)\varphi \left(\rho ,\nu \right)\;dr$$ 20

The function φ­(ρ, ν) introducing the dependence on ν was adjusted to the analytical form

21 $$\varphi \left(\rho ,\nu \right)\approx {\left(\frac{\nu }{n_{+}\left(r_{s}\right)}\right)}^{\gamma }$$

for ν < *n*_+_(*r*_s_), where the γ value was fitted to 0.329 by using the momentum distribution from Paijanne and Arponen^49^ and coupled-cluster calculations from Freeman.^50^ For ν > *n*_+_(*r*_s_), φ­(ρ, ν) can be fixed to 1 by imposing ε_c_(ρ)­φ­(ρ, ν) ≤ ε_c_(ρ), ∀ *r*. Hence, for large ν, eq 20 recovers the full DFT correlation, whereas for ν → 0, the DFT correlation energy vanishes.

### 3.3 Sampling the Orbital Occupation Space

The WFT-*so*DFT strategy relies on having access to an accurate profile of the NOONs of the system. Therefore, the WFA should be able to produce a distribution close to the exact one, in particular, in the small-occupation region, which is the one eventually dictating the *so*DFT correlation energy through the ν parameter. Hence, the use of compact multiconfigurational approaches (featuring a very small set of configurations) to generate NOs and their NOONs, although they could provide a good approximation of ρ, might be insufficient for the accurate assessment of *E*_c_^*so*,ν^[ρ]. Similarly, small basis sets might not provide the flexibility necessary to characterize *n*(*k* > *k*_F_, *r*_s_). On the other hand, employing highly correlated wave function approaches, e.g., CASSCF with a large active space, would inevitably increase the computational demands of the method and defeat the purpose of the WFT-*so*DFT strategy. Therefore, in principle, an ideal ansatz should present two important features: (i) a small number of configurations capable of recovering the system-dependent features (strong correlations) and (ii) a limited set of additional terms able to describe the distribution of small-occupation NOONs.

Here we propose the restricted active-space CI (RASCI)^2^ method within the one-*hole* and one-*particle* approximation as implemented in the Q-Chem package.^51,52^ The single-reference RASCI family of methods emerges from splitting the orbital space of a reference configuration (ϕ_0_) into three different subspaces: RAS1, RAS2, and RAS3, with RAS1 (RAS3) orbitals being doubly occupied (virtual) in ϕ_0_. The RASCI wave function for a target state (|Ψ⟩) is obtained by the action of an excitation operator (R̂) on the reference configuration as

22 $$|\Psi \rangle =\mathrm{R̂}|\phi_{0}\rangle$$

where the operator *R̂* is expanded as

23 $$\mathrm{R̂}={\mathrm{r̂}}_{0}+{\mathrm{r̂}}_{h}+{\mathrm{r̂}}_{p}+{\mathrm{r̂}}_{hp}+{\mathrm{r̂}}_{2h}+{\mathrm{r̂}}_{2p}...$$

with ${\mathrm{r̂}}_{0}$ containing all possible excitations within RAS2 and the rest of the terms in eq 23 generating configurations with increasing number of holes (*h* subindex) in RAS1 and/or particles (*p* subindex) in RAS3.

In order to achieve the two indicated requirements of WFAs for the generation of the NOON distribution, we consider RASCI wave functions with a small RAS2 space (to capture strong correlations) and the truncation of the excitation operator to $\mathrm{R̂}={\mathrm{r̂}}_{0}+{\mathrm{r̂}}_{h}+{\mathrm{r̂}}_{p}$ , that is, the *hole* and *particle* approach [RASCI­(*h*, *p*)], as schematically shown in Figure 2.

**Figure 2:**
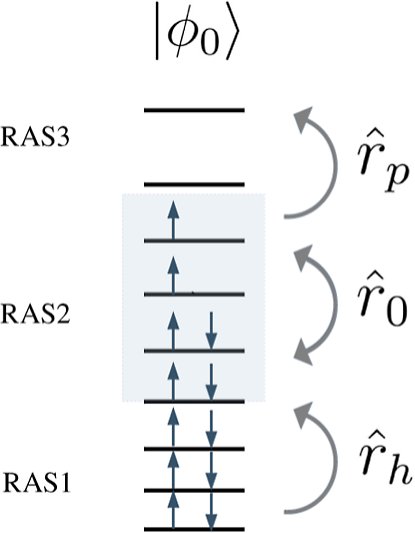
Representation of the three RAS orbital spaces and the action of the ${\mathrm{r̂}}_{0}$ , ${\mathrm{r̂}}_{h}$ (*hole*), and ${\mathrm{r̂}}_{p}$ (*particle*) terms of the excitation operator on the reference state (|ϕ_0_⟩).

To exemplify how the chosen ansatz is able to evaluate the distribution of NOONs, we compute the ground-state NOs and NOONs for the all-*trans* octatetraene (C_8_H_10_) with different truncations of *R̂*. In this case, pristine RASCI wave functions (with no *so*DFT contribution) were obtained by applying a quadruple spin-flip (SF) operator to the high-spin (*S* = 4) ROHF reference. The RAS2 space was defined by the 8 electrons in the 8 singly occupied π-orbitals in the ROHF reference, with RAS1 and RAS3 including all doubly occupied and doubly unoccupied orbitals, respectively. The molecular geometry was optimized at the MP2/6-311­(2+,2+)­G­(d,p) computational level.^53^ Similar qualitative results were obtained for other molecules (see Supporting Information).

Figure 3a shows the profiles of the low-occupation tail of ground-state NOONs computed with $\mathrm{R̂}={\mathrm{r̂}}_{0}$ [RASCI(0)], $\mathrm{R̂}={\mathrm{r̂}}_{0}+{\mathrm{r̂}}_{h}$ [RASCI­(*h*)], $\mathrm{R̂}={\mathrm{r̂}}_{0}+{\mathrm{r̂}}_{p}$ [RASCI­(*p*)], and $\mathrm{R̂}={\mathrm{r̂}}_{0}+{\mathrm{r̂}}_{h}+{\mathrm{r̂}}_{p}$ [RASCI­(*h*, *p*)]. The RASCI(0) truncation, i.e., CASCI, is unable to populate the orbital space beyond RAS2. It produces a set of NOs (with the dimension of RAS3) with vanishing NOONs. Hence, this approach, like CASSCF with small and moderate active spaces, cannot be used as a WFA in WFT-*so*DFT. Including *hole* excitations does not provide any change in the small-occupation region. This is expected since RASCI­(*h*) only allows orbital mixing between RAS1 and RAS2 spaces and can only generate fractional NOONs in the large-occupation region and the strongly correlated space (RAS2). On the other hand, allowing excitations into the (much larger) RAS3 space, RASCI­(*p*), produces a finite distribution of NOs with small NOONs. Therefore, we conclude that the presence of *particle* terms is mandatory. In this example, allowing both types of excitations does not involve any appreciable change in the description of *n*_+_(*k* > *k*_F_, *r*_s_) with respect to RASCI­(*p*), but the simultaneous presence of both types of excitation allows the mixing of the entire orbital space (RAS1 + RAS2 + RAS3). Moreover, the computational cost associated with *hole* excitations is, in general, considerably smaller than that related to *particle* configurations. In other words, the size of RAS3 (proportional to the number of basis set functions) is much larger than RAS1 (proportional to the system size). Therefore, RASCI­(*h*, *p*) will be the WFA used throughout the rest of the study.

**Figure 3:**
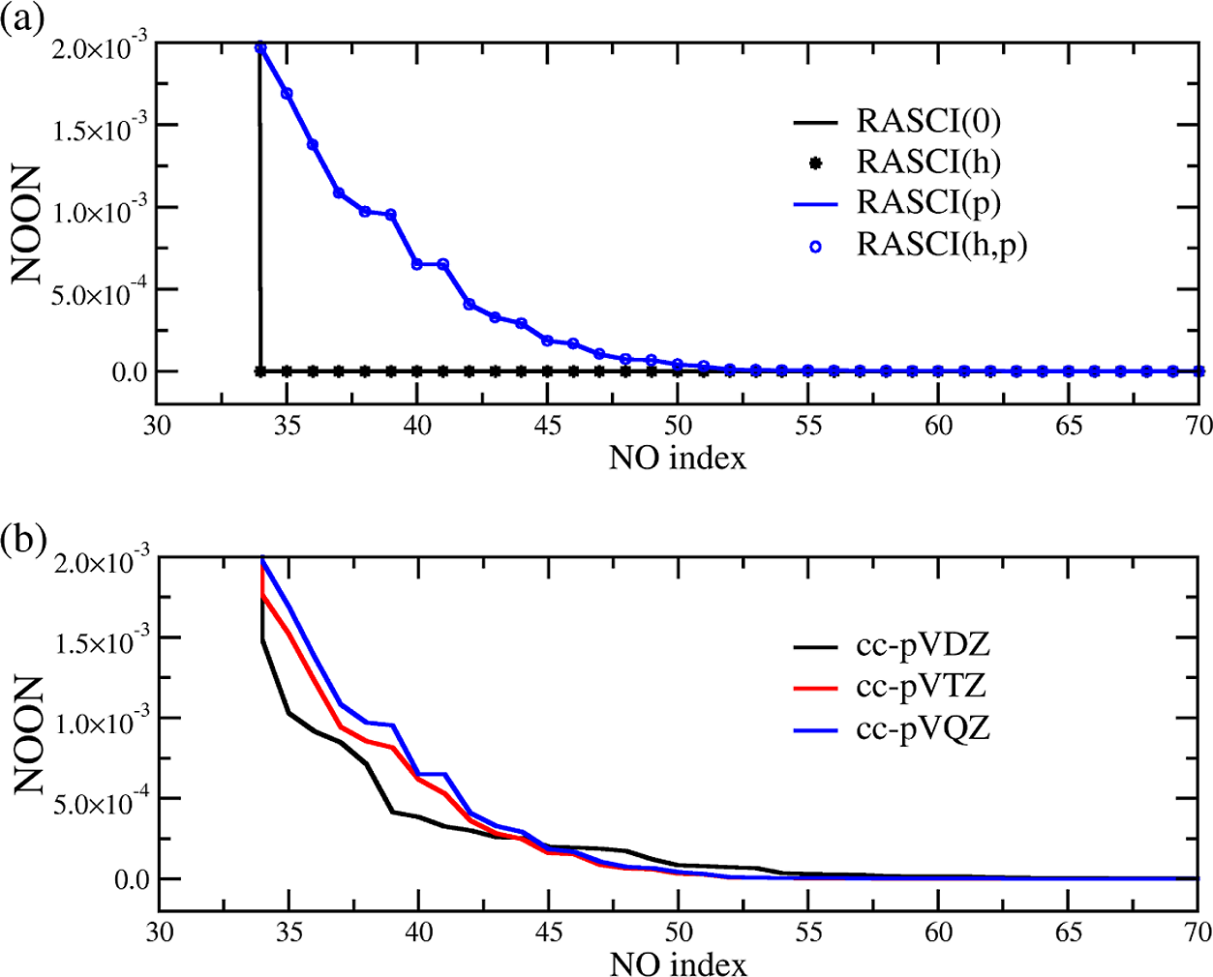
Ground-state NOONs at the tail of the NO space of octatetraene computed with the (a) cc-pVQZ basis and different truncations of *R̂* and (b) RASCI­(*h*, *p*)/cc-pVXZ with X = D, T, and Q.

The importance of *particle* excitations in the sampling of *n*_+_(*k* > *k*_F_, *r*_s_) is directly related to the basis set employed, as it defines the size and properties of the RAS3 space. Figure 3b shows the small-occupation profiles for the ground state of octatetraene obtained with RASCI­(*h*, *p*) and with the cc-pVXZ (X = D, T, and Q) series of basis. Increasing the size of the basis set modifies the profile of the NOON distribution, with an overall electron occupation of the NOs with the smallest occupations (integration of the NOONs of NOs beyond the RAS2 space) increasing with the dimensions of the basis set: 8.3 × 10^–3^ (cc-pVDZ), 9.8 × 10^–3^ (cc-pVTZ), and 11.1 × 10^–3^ (cc-pVQZ) electrons. Therefore, as desired, increasing the size of the basis set will trigger a larger *so*DFT correlation correction energy into WFA.

## 4 Methods

### 4.1 Computational Workflow

The general procedure for computation of the WFT-*so*DFT energy can be schematically described as follows:

1. Initial WFA calculation to obtain NO and NOON pairs (eq 2).
2. Select occupation-separation parameter ν (eq 12 or 13).
3. Compute *so*DFT correlation energy: *E*_c_^*so*,ν^[ρ] (eq 20).
4. Compute the ν-dependent WFA energy: $E_{0}^{WFA,\nu }$ = $\min_{\Psi^{\nu }}\left\langle \Psi^{\nu }\left|Ĥ\right|\Psi^{\nu }\right\rangle$ (eq 11).
5. Obtain the total energy by adding both contributions (eq 10).

Note that the *so*DFT correlation energy (step 3) is computed by employing the electron density obtained from the initial WFA calculation (step 1). This is justified by the fact that, in general, the initial WFA is designed to provide good NO and NOON pairs, i.e., with an electron density expected to mildly deviate from the exact one (assumption in eq 8). The ν-dependent WFA energy (step 4) is computed with the chosen WFA but disregarding the NOs with NOONs smaller than the ν parameter.

### 4.2 Computational Details and Nomenclature

All WFT calculations were done with the RASCI­(*h*, *p*) methodology with the RAS2 space specifically selected for each case and with RAS1 (RAS3) including all orbitals doubly occupied (unoccupied) in the reference configuration (not included in RAS2). The nature of the excitation operator *R̂* in RASCI­(*h*, *p*):^53,54^ excitation energy (EE), SF, ionization potential (IP), or electron attachment (EA), is specified in each example (see Section 5). Computation of Ψ^ν^ in step 4 was obtained by removing those NOs with NOONs smaller than ν. Unless indicated, the dimension of RAS2 used to obtain *E* ^WFA,ν^ was the same as the one employed in the initial WFA calculation. In practice, the computation of the DFT correlation energy has to rely on an approximation, i.e., density function approximation (DFA). Here we employ the Vosko, Wilk, and Nusair (VWN) LDA correlation functional.^55^ Possible extensions to other LDA or generalized gradient approximation (GGA) correlation functionals or the use of fictitious spin density to mimic the effects of unpaired electrons (as in spin-density functionals) has not been explored in the present work. The introduced methodology, with RASCI­(*h*, *p*) as the WFA and *so*VWN as the small-occupation correlation energy functional, will be labeled as RAS-*so*VWN. When necessary, the *S* or *G* labels will be used to explicitly indicate Savin’s [RAS-*so*VWN­(S), eq 18] or Gori-Giorgi’s [RAS-*so*VWN­(G), eq 19] form of *n*_+_(*r*_s_), respectively. In order to evaluate the impact of the (potential) double counting of correlation effects, for some of the systems studied in Section 5, we explore the total electronic energy obtained by the simple addition of the *so*DFT correlation energy to the pristine WFA energy (eq 2), that is, skipping step 4 in the computational workflow. These calculations can be found in the Supporting Information. Unless indicated, the selection of the occupation-separation parameter ν has been done by taking into account all small-occupation NOONs (eq 13) by setting a threshold to the integral on the LHS of eq 13 (equivalent to fixing *k*_c_). Results obtained with the use of a single NOON instead (eq 12) are shown in the Supporting Information. The computation of $E_{0}^{WFA,\nu }$ (eq 11) is performed at the RASCI level in the NO basis with the same RAS1 and RAS2 dimensions set for the initial RASCI calculation of NOs and their NOONs. In order to ensure that strong correlations are dealt with by WFT, the minimum orbital dimension included in the WFA contribution, i.e., the number of NOs, is never smaller than RAS1 + RAS2 space (even if the ν threshold has not been reached). The analysis of the dependence of the *so*DFT energy with respect to parameter ν can be found in the Supporting Information.

Configuration interaction using a perturbative selection done iteratively (CIPSI) calculations have been performed with the Quantum package by setting a maximum of two million determinants entering in the internal space and PT2 energy threshold below 10^–6^ a.u.^56^ All other electronic structure calculations have been carried out with the Q-Chem package.^57^ The WFT-*so*DFT approach has been implemented in combination with RASCI wave functions in a development version of Q-Chem.

## 5 Results and Discussion

### 5.1 Helium and Beryllium Atomic Series

The He and Be isoelectronic series, consisting of atoms and ions with the same electron count but varying nuclear charges (*Z*), play a pivotal role in evaluating electronic structure methods and have been widely used to calibrate dynamic and nondynamic correlation effects.^58,59^ As *Z* increases, dimensional arguments suggest that the radius of the electron cloud decreases as *Z* ^–1^, the electron kinetic energy increases as *Z* ^2^, the electron–nuclear potential energy is proportional to −*Z* ^2^, and the electron–electron potential energy (the Hartree part) is linear with *Z*.^60^ Therefore, the one-body part of the Hamiltonian dominates the electron–electron interaction, and the correlation energy should be amenable to a perturbative treatment.

In the He series, the ground state remains energetically isolated up to a large *Z*, suggesting that dynamic correlation effects are far more important than the nondynamic correlations (somehow related to degeneracies). Perturbation theory indicates that the correlation energy converges to a constant value at the *Z* → ∞ limit. In this case, LDA overestimates correlation energies that increase with *Z*, yielding 3.0 eV for He and 5.5 eV for Ne^8+^,^32^ while the anticipated values should remain around 1 eV for the entire series.^32,61^ Electron density increases with *Z*, as a consequence, for larger values of *Z*, LDA samples the correlation energy of very dense (*r*_s_ → 0) electron gases, where the continuous energy level spacing and long-ranged Coulomb interaction trigger a ln *r*_s_ divergence of the energy density.^62^ On the other hand, in the (neutral) Be atom, the 2s and 2p orbitals are nearly degenerate (quite small HOMO–LUMO gap). As *Z* increases with the number of electrons fixed to 4, the orbital gap increases but the relative gap (the gap divided by the average energy of the orbitals) actually decreases (type B of nondynamic correlation).^63^ For *Z* → ∞, *E*_c_ diverges linearly. This feature is not reproduced by standard correlation density functionals, e.g., *E*_c_^LDA^ ∼ ln *Z*.^64^ In the exact perturbative treatment, the energy gap diminishes swiftly with the spacing of energy levels in the denominator playing a pivotal role in determining the correlation energy.

In order to check the performance of our RAS-*so*DFT approach, we compute the electronic energies for these two atomic series. Figure 4 shows the errors of RASCI­(*h*, *p*) and RAS-*so*VWN with respect to FCI energies.^65^ All calculations have been done with an RAS2 space including 5 orbitals and all electrons (2 in the He series and 4 in the Be series) and the cc-pVQZ basis set.

**Figure 4:**
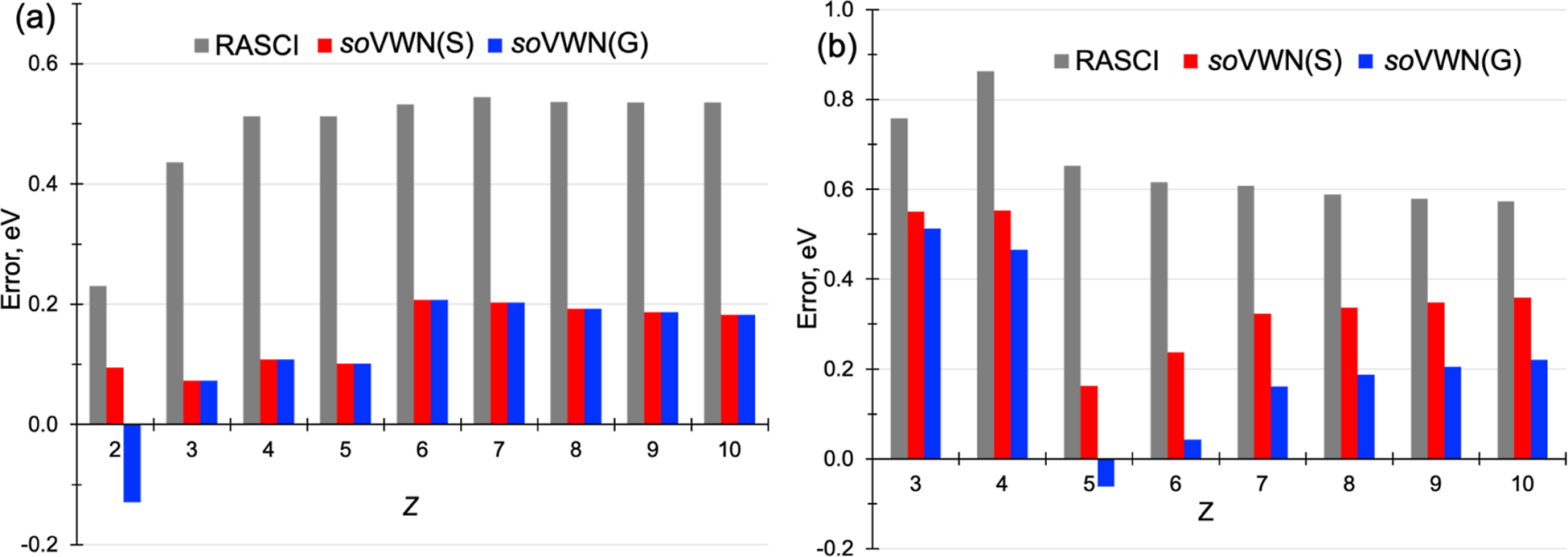
RASCI­(*h*, *p*) and RAS-*so*VWN (labeled as *so*VWN with ν = 0.001) energy errors (in eV) with respect to FCI values computed with the cc-pVQZ basis set for the He (a) and Be (b) atomic series. *S* and *G* in parentheses indicate the two considered expressions for *n*_+_(*r*_s_), eqs 18 and 19, respectively.

Despite their simplicity as two- and four-electron systems, both series present significant challenges for RASCI­(*h*, *p*). Even with the inclusion of five orbitals in RAS2 (1s, 2s, and 2p orbitals), the discrepancies relative to FCI are substantial, on the order of 0.5 eV (approximately 0.2 eV for He) along the He series (see Figure 4a) and even larger (0.6–0.9 eV) in the Be series (see Figure 4b). RAS-*so*VWN consistently and significantly mitigates these discrepancies. Remarkably, within the two-electron series, with the exception of the He atom, the electron density predominantly resides within the first five NOs, resulting in almost negligible *so*DFT correlation energies (a vanishing tail of small-occupation NOONs as shown in Figure S2). As a consequence, the enhancement in the correlation energies is directly linked to $E_{0}^{WFA,\nu }$ , which reflects the utilization of NOs in the variational optimization of the ground-state wave function. This pattern holds true except for the He atom, where a substantial contribution from the DFT correlation correction is observed. This contribution becomes more pronounced when employing the *n*_+_(*r*_s_) function proposed by Gori (eq 19). The scenario is quite distinct in the Be series (Figure S3), where there is little to no enhancement observed in $E_{0}^{WFA,\nu }$ compared to RASCI (Figure S5). However, a substantial correction arises from the *so*DFT correlation in the atomic energies, with notably significant effects for *Z* ≥ 5.

### 5.2 H_2_ Dissociation

A bond breaking/formation process can be described as a continuous closed-to open-shell transition. The σ-bond dissociation in H_2_ is the simplest and most paradigmatic example of such a transition. As the molecule is stretched, the gap between the σ and σ* orbitals, HOMO and LUMO, decreases, facilitating the electron population of the σ* orbital. As a consequence, the singlet ground-state wave function is no longer well-described with a single configuration.^66,67^ Hence, a method able to describe the H_2_ potential energy surface (PES) in a balanced manner should treat dynamic correlation between the two electrons (important at equilibrium) and nondynamic correlation (crucial at long distances).

Here, we assess the performance of our *so*DFT correction on the RAS-SF/cc-pVDZ energies computed for the H_2_ dissociation process. We utilize the ROHF triplet as the reference configuration and an RAS2 space with 2 electrons in 2 orbitals. The pristine wave function solution yields a smooth energy profile, converging to the exact (FCI) solution at large interatomic separations, albeit with a slight overestimation of the electronic energy at equilibrium (see Figure 5a). Therefore, our aim is to have the *so*DFT approach recover the missing correlation energy at short distances while approaching zero at large distances. Indeed, the NOONs at equilibrium display a tail of small occupancies, contributing to an *so*DFT energy that leads to a lower overall RAS-*so*VWN energy (closer to the FCI solution). Conversely, as the bond distance increases, the spectral resolution of the density matrix evolves toward two singly occupied NOs (with no small-occupation tail), i.e., *E*_c_^*so*,ν^ → 0 (see Figure 5b,c). Therefore, overall, RAS-*so*VWN improves the RAS-SF results by incorporating electron correlation effects at short distances. We note that at very short interatomic separation (<0.7 Å), the *so*DFT correlation ill-behaves (not shown in Figure 5c). Complete profiles for the sum of NOONs and *E*_c_^*so*,ν^ can be found in the Supporting Information (Figure S6).

**Figure 5:**
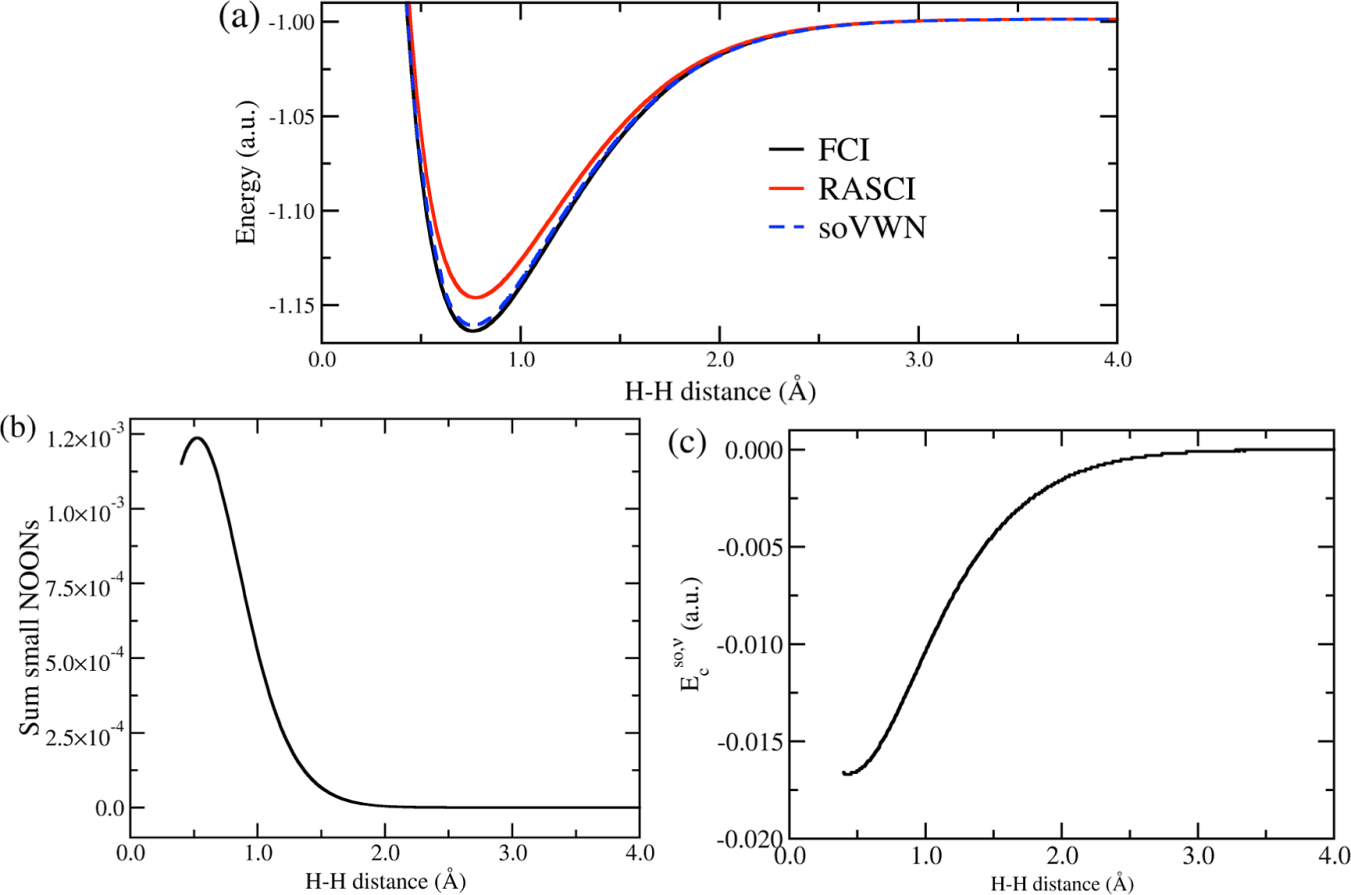
(a) Total electronic energy profiles (in a.u.), (b) sum of small NOONs, and (c) RAS-*so*VWN (*so*VWN in short) energy (in a.u.) along the H_2_ dissociation.

The improvement in the calculated relative energy between the closed and open electronic structures becomes evident with the computed dissociation energy, as shown in Table 1. At the equilibrium distance, the absence of dynamic correlation in RAS-SF leads to a significant underestimation of the binding energy compared to FCI, notably corrected by RAS-*so*VWN. It is noteworthy that, in this case, the wave function energy contribution to RAS-*so*VWN $\left(E_{0}^{WFA,\nu }\right)$ yields nearly identical values to those obtained with RAS-SF, indicating that the *so*VWN term (*E*_c_^*so*,ν^) primarily drives the increase in the computed dissociation energies. In this example, the most favorable outcome is achieved with a threshold of ν = 0.01 and employing eq 19 for the distribution of the NOONs of the HEG. Larger values, i.e., ν = 0.1, tend to overestimate the correlation energy from NOs with low occupation, while ν ≤ 0.001 for RAS-*so*VWN­(S) and smaller for RAS-*so*VWN­(G) lead to vanishing *E*_c_^*so*,ν^.

**Table 1:** Dissociation Energies (in mhartrees) for H_2_ within the cc-pVDZ Basis Set^a^

method	ν-threshold		
	0.1	0.01	0.001
*so*VWN(S)	177.3	156.6	148.6
*so*VWN(G)	192.7	162.2	162.2
$E_{0}^{WFA,\nu }$	145.9	148.5	148.5
RAS-SF	141.3		
FCI	163.9		
Exp.^b^	164.5		

a These results illustrate three different partitions of the NO space defined through the values for the threshold. $E_{0}^{WFA,\nu }$ wavefunction contribution defined in eq 11.

b Experimental value taken from ref 68.

### 5.3 Ethylene Torsion

Double-bond torsion in ethylene represents the simplest molecular model of single π-bond breaking. At equilibrium geometry, the two carbon p_*z*_ orbitals, perpendicular to the molecular plane, form the bonding π and antibonding π* orbitals. Departure from the planar (*D*_2*h*_) structure through torsion between the two methylene moieties diminishes the overlap and the interaction between the two p orbitals that become orthogonal at 90° of torsion (zero overlap). At the perpendicular disposition, the two frontier orbitals are degenerated and the system exhibits a perfect diradical character. As pointed out in prior works, the accurate description of the torsional potential claims for a balanced treatment of electron correlation effects.^69–76^

Single-reference methods struggle to describe the electronic structure for large dihedral angles. At 90° torsion, the wave function should include the (π)^2^ and ${\left(\pi^{*}\right)}^{2}$ configurations treated on an equal footing. However, the ${\left(\pi^{*}\right)}^{2}$ configuration is completely neglected by the restricted HF (RHF) model. As a result, RHF produces a potential curve with an unphysical cusp and a large overestimation of the barrier height (101.5 kcal/mol) compared with the ∼65 kcal/mol estimated from experimental data^77^ or by multiconfigurational correlation methods^78^ (Table 2). This failure of the mean-field solution is carried over to post-HF wave functions such as MP2, CISD, or CCSD or even in (restricted) Kohn–Sham approximations to DFT.^71^ Energy profiles are notably improved by spin-polarized (unrestricted) schemes, by multireference wave functions, e.g., CASSCF,^76^ or by the use of SF methods in combination with a high-spin (triplet) reference configuration.^74^

**Table 2:** Energy Barrier (kcal/mol) Computed for the Molecular Torsion of Ethylene^a^

method	barrier
RAS-SF	54.5
*so*VWN (0.01)	62.24
*so*VWN (0.001)	66.71
*so*VWN (0.0001)	67.23
RHF	109.0
B3LYP	95.3^b^
CASSCF	57.1
VOO-CCD	67.3^c^
MRDCI	62.7^c^

a RAS-*so*VWN (indicated as *so*VWN), with values in parentheses corresponding to the employed sum of occupations threshold.

b Value reported in Computational Chemistry Comparison and Benchmark Data Base Release 22, May 2022 (https://cccbdb.nist.gov), with the 6-31G­(d) basis set.

c From ref 71, with the DZP basis set.

In this study, we conduct calculations along the twist mode of ethylene while keeping all other degrees of freedom frozen at the ground-state (singlet) geometry, which was optimized at the CCSD­(T)/cc-pVDZ level.^79^ RASCI calculations were performed using an SF operator acting on the ROHF triplet state, employing an RAS2 space with 8 electrons distributed over 7 orbitals and utilizing the cc-pVDZ basis set. The potential energy curves along the torsional coordinate are presented in Figure S10. The RAS-SF wave function yields a smooth energy profile, demonstrating good agreement with prior studies.^71,76,77^ Notably, the energy gap between the planar and orthogonal conformations (54.5 kcal/mol) avoids the substantial overestimation observed in the (restricted) HF and B3LYP calculations. However, it does exhibit a slight underestimation of the torsion barrier by approximately 10 kcal/mol when compared to values obtained with highly correlated methods (VOO-CCD and MRCI).^71,78^ This underestimation is considerably ameliorated by the *so*DFT correlation, which introduces a differential correlation at the planar structure compared to the barrier maximum (twisted geometry). This improvement appears to be robust with respect to the chosen ν threshold, particularly in the range of 10^–3^ to 10^–4^ (values below 10^–4^ result in negligible *so*DFT correction). This suggests a counterbalancing effect between the WFA and *so*DFT contributions.

### 5.4 Multiple Bond Breaking: N_2_ Dissociation

The dissociation of N_2_ involves a multistep bond-breaking process, transitioning from the ground-state electronic configuration at the minimum (σ_g_^2^π_u_^4^π_g_^0^σ_u_^0^) to the dissociation limit, ultimately yielding two spin-quartet fragments. The accurate description of the correlation energy in this diatomic system has been revisited several times, in many cases for the purpose of evaluating the performance of correlated quantum chemical methods.^80,81^ Several challenges arise in the theoretical depiction of N_2_ dissociation, depending on the chosen method. These include inconsistencies in the CI expansion,^82^ accounting for valence-core electron correlations,^83^ or the effect of basis functions with high angular momenta (e.g., *g*, *h*, and *i* functions).^84^

In the subsequent discussion, we delve into the characteristics of the PES concerning N_2_ dissociation. This exploration is conducted with the RASCI­(*h*, *p*) and RAS-*so*DFT computational approaches, employing an RHF singlet reference and incorporating six electrons and six frontier orbitals (σ, π, σ*, and π*) as the RAS2 space, in conjunction with the cc-pVDZ basis set. The computed total electronic energy profiles for the stretching of N_2_ are depicted in Figure 6a. While the RASCI profile exhibits smooth behavior, the lack of effective dynamic correlation leads to energies significantly higher than those of the more correlated CIPSI solution. Analysis of the WFA contribution $\left(E_{0}^{WFA,\nu }\right)$ reveals that the utilization of NOs imparts only a marginal effect around the bond equilibrium distance, thereby adding correlation energy relative to RASCI. In contrast, the DFT-corrected RAS-*so*VWN energies, obtained with a ν-threshold value of 0.001, are energetically lower (by approximately 50 mhartrees), that is, closer to the CIPSI curve, but the energy profile presents several (many) discontinuous steps. This issue emerges solely from the *so*DFT contribution (Figure 6b, red line), while the $E_{0}^{WFA,\nu }$ has a smooth profile. The analysis of the small electron occupation entering the *so*DFT (Figure 6b, black line) indicates that the sudden changes in *E*_c_^*so*,ν^ are linked to the discontinuous distribution of NOs. Concretely, the steps in the RAS-*so*VWN energy curve occur at interatomic distances in which the number of NOs included in the *so*DFT part changes (an addition of one NO when the bond distance increases). This change implies a discontinuous “jump” in the ν value employed for the evaluation of *E*_c_^*so*,ν^ (eq 21). Fixing the number of NOs included in *so*DFT to a constant value (instead of the parameter ν) represents a partial solution to this artifact (Figure S20). These results highlight an important drawback of the method in the characterization of PESs.

**Figure 6:**
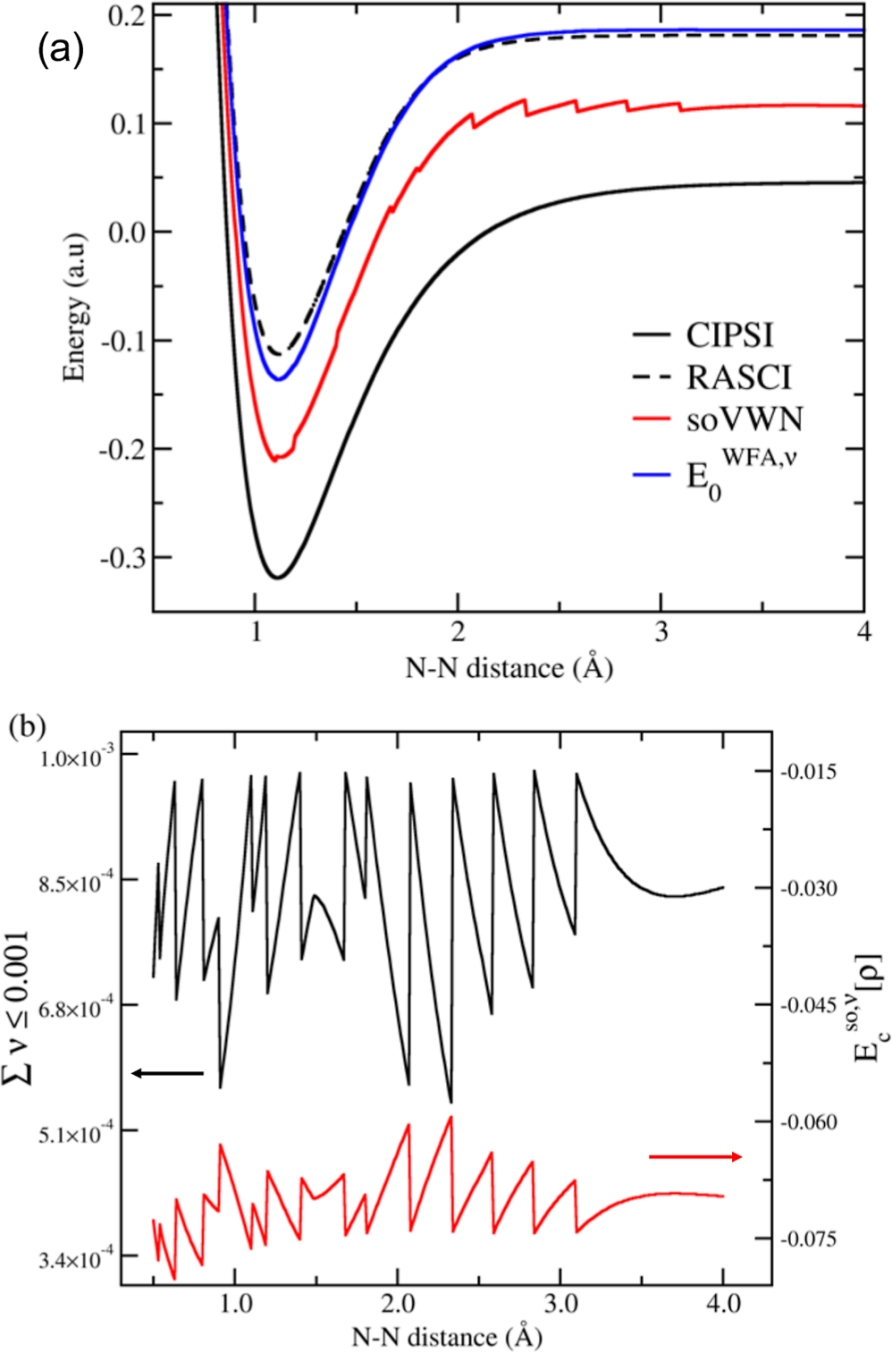
(a) Absolute energy profiles along the N_2_ dissociation computed at the RASCI (dashed black), RAS-*so*VWN (ν = 0.001, solid red), $E_{0}^{WFA,\nu }$ contribution (solid blue), and CIPSI (solid black) computed with the cc-pVDZ basis set. (b) Sum of NOONs below the threshold ν = 0.001 (black) and *E*_c_^*so*,ν^ (red) along the N_2_ dissociation.

Dissociation energies (as listed in Table 3) are computed by taking the difference between twice the energy of a nitrogen atom (with 3 electrons distributed across 3 orbitals within the RAS2 space) and the energy of N_2_ at its equilibrium bond distance. As observed in the cases of H_2_ dissociation and ethylene torsion, RASCI notably underestimates the binding energy of N_2_ in comparison to highly accurate methods and experimental data.^68^ The use of ground-state energies computed with NOs having NOONs above the ν threshold already leads to improved results, yielding dissociation energies similar to those obtained from CASSCF. Furthermore, the inclusion of the *so*DFT correlation significantly enhances the computed binding energy, bringing it closer to values obtained from MRCI and experimental measurements, particularly for ν thresholds in the range of 0.001 to 0.0001. It is worth noting that larger ν values tend to overestimate the energy difference between N_2_ at equilibrium and twice the atomic energy.

**Table 3:** N_2_ Dissociation Energies (in kcal/mol) Computed with CASSCF, RASCI, $E_{0}^{WFA,\nu }$ , and RAS-*so*VWN (*so*VWN in Short) with the cc-pVDZ and Compared to Reference Results from the Literature

method	ν threshold			
	0.1	0.01	0.001	0.0001
$E_{0}^{WFA,\nu }$	193.3	193.3	197.7	202.6
*so*VWN(S)	250.6	250.6	230.0	202.6
*so*VWN(G)	276.2	282.6	243.6	202.6
RASCI	185.4			
CASSCF^a^	196.6			
NEVPT2^b^	201.2			
CIPSI	201.4			
MRCI^c^	222.1			
Exp^d^	225.1			

a Active space with 6 electrons in 6 orbitals and the cc-pVDZ basis.

b From refs 85–87.

c From ref 80, with the [5s 4p 3d 2f lg] ANO basis set.

d From ref 68.

## 6 Conclusions

In this work, we have introduced a novel WFT-DFT hybrid approach designed to incorporate DFT correlation into wave function ansatzes by partitioning the orbital space into large and small NOONs, respectively, assigned to WFT and DFT correlation. The new method (WFT-*so*DFT) is based on previous ideas from Savin, but it incorporates different criteria for the splitting of the orbital space and the characterization of the small-occupation tail of the HEG distribution. Concretely, we have considered the use of the RASCI wave function approach within the *hole* and *particle* truncation, which provides a simple and rather efficient strategy to sample the space of small-occupation NOs necessary to construct the small-occupation correlation energy functional. Besides the selection of the WFA and correlation functional, the method relies on a separation parameter ν. The preliminary results here discussed seem to indicate that low ν values (between 10^–3^ and 10^–4^) can provide reasonable correlation energies for the correction of multiconfiguration wave functions of the ground state of medium-size molecules. However, systematic benchmarking is still necessary to better understand the dependence and universality of the occupation-separation parameter. Moreover, the comparison of WFT-*so*DFT with other WFT-DFT hybrid models, e.g., WFT-*sr*DFT or MC-PDFT, and with multiconfigurational perturbation theory remains to be explored. These efforts are expected to be carried out in future studies. Furthermore, since the method requires a good description of the small-occupation region of the electron density, it is prone to be used with large basis sets. The results obtained by RAS-*so*VWN in the computation of atomic energies along the He and Be series, the dissociation of single (H_2_) and multiple (N_2_) bonds, and the torsion of ethylene improve those obtained by the pristine wave function (RASCI). In some cases, the use of NOs in the WFA already represents an important refinement of the initial wave function, while correlation correction might be important in some situations, e.g., as seen at equilibrium geometries, and insignificant in others, like for the He series with *Z* > 2. The discrete distribution of NOONs obtained with finite basis sets might trigger discontinuities in PESs, as seen in the calculation of N_2_ dissociation. Despite the fact that this unphysical effect can be partially avoided by fixing the number of NOs in *so*DFT, it represents an important limitation of the method for the characterization of large domains of PESs.

## Supplementary Material



## Data Availability

The data that supports the findings of this study are available within the article and its Supporting Information, which contains additional and complementary data for the studied systems.

## Notes

Additional information for polyene systems, numerical results for atomic series, H_2_ bond dissociation, C_2_H_4_ molecular torsion, N_2_ triple bond dissociation, and size consistency analysis (PDF)
