# Supplementary material for: Small-Occupation Density Functional Correlation Energy Correction to Wave Function Approximations
Source: J Chem Theory Comput. 2024 Jan 16;20(3):1118–29. doi: 10.1021/acs.jctc.3c01067 (PMC12865771; doi:10.1021/acs.jctc.3c01067)
Supplement: Supplementary file 1 [file ct3c01067_si_001.pdf]

Supporting Information: Small-occupation density functional correlation energy correction to wavefunction approximations

José Aarón Rodríguez-Jiménez,[†,¶] Abel Carreras,[‡] and David Casanova[*,†,§]

†*Donostia International Physics Center (DIPC), 20018 Donostia, Euskadi, Spain.*

‡*Multiverse Computing, 20008 Donostia, Euskadi, Spain.*

¶*Polimero eta Material Aurreratuak: Fisika, Kimika eta Teknologia, Kimika Fakultatea, Euskal Herriko Unibertsitatea (UPV/EHU), 20018 Donostia, Euskadi, Spain.*

§*IKERBASQUE, Basque Foundation for Science, 48009 Bilbao, Euskadi, Spain.*

E-mail: david.casanova@dipc.org

Contents

1 Additional information for polyene systems **S3**

2 Numerical results for atomic series **S5**

2.1 Complementary data for Atomic series S5

2.2 Performance of different variants of the method S6

2.3 Comparison of correlation energies computed for the Be-series. S7

2.4 Total energies computed with the cc-pVQZ basis set S8

3 H_2 bond dissociation **S10**

3.1 Complementary data for H_2 dissociation S10

3.2 Potential energy surfaces for H_2 . . . . S10

3.3 Energy dependence on ν parameter S14

3.4 Basis set dependence S15

4 C_2H_4 molecular torsion **S16**

4.1 Complementary data for C_2H_4 torsion S16

4.2 Potential energy surfaces for C_2H_4 . . . . S16

4.3 Energy dependence on ν parameter S19

5 N_2 triple bond dissociation **S20**

5.1 RASCI Natural orbital occupations computed for the N_2 . . . . S20

5.2 Potential energy surfaces for N_2 . . . . S21

5.3 Energy dependence on ν parameter S29

5.4 Reducing discontinuities S30

6 Size consistency analysis **S30**

References **S34**

1 Additional information for polyene systems

We use the linear n-polyenes ($C_{2n}H_{2n+1}$, $n = 2-4$), to show the changes in NOON distribution affected by system size and level of theory. From considerations previously mentioned, in order to perform a correct orbital splitting and to incorporate DFT correlation, the distribution of occupations should behave similar to the momentum distribution for the HEG. Additionally, since the splitting of the NO space occurs at RAS3, it is fundamental that this subspace must be occupied (on the contrary, no DFT energy contribution is possible). Here we illustrate such characteristics through the use of CASCI and RASCI methodologies. To compute electronic wavefunctions through n spin-flip excitations from the ROHF single reference with $2n+1$ spin multiplicity, i.e. single alpha-occupation for the $2n$ frontier pi-orbitals (RAS-nSF) the RAS2 space for each case is naturally defined as $2n$ electrons in the $2n$ pi-orbitals. Geometries have been optimized for the singlet ground state at the MP2/6-311(2+,2+)G(d,p) computational level.[S1]

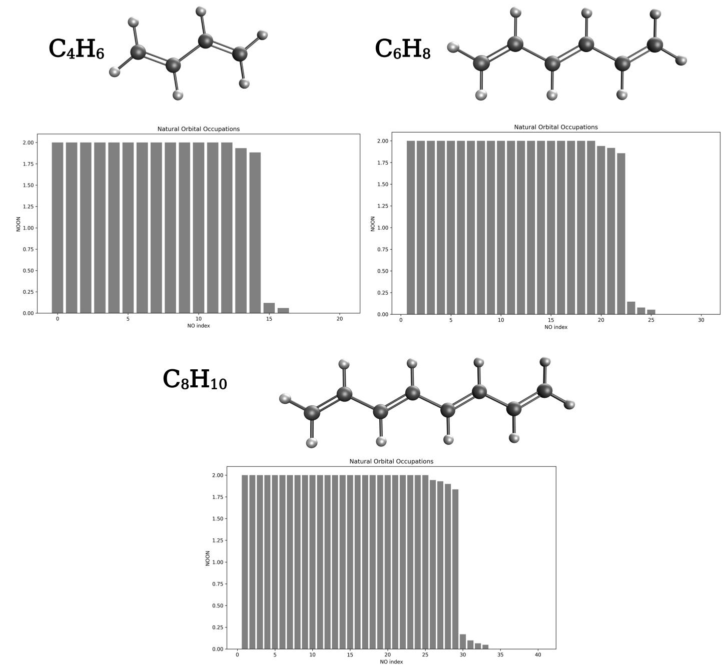

Figure S1: Illustrative NOONs computed at the RASCI(h,p)/cc-pVQZ level for $C_{2n}H_{2n+1}$ linear polyenes (n = 2-4).

Table S1: Sum of NO occupations for the ground singlet state ($1^1A_g^-$) in each RAS subspace for linear n-polyenes ($C_{2n}H_{2n+1}$).

basis	RAS1	RAS2	RAS3
	2-polyene		
cc-pVDZ	25.9960	4.0002	0.0039
cc-pVTZ	26.0000	3.9970	0.0030
cc-pVQZ	26.0000	3.9965	0.0035
	3-polyene		
cc-pVDZ	37.9930	6.0006	0.0064
cc-pVTZ	38.0000	5.9936	0.0064
cc-pVQZ	38.0000	5.9927	0.0073
	4-polyene		
cc-pVDZ	49.9953	7.9963	0.0083
cc-pVTZ	50.0000	7.9902	0.0098
cc-pVQZ	50.0000	7.9889	0.0111

Table S2: Sum of NO occupations for the ground singlet state ($1^1A_g^-$) in each RAS subspace for linear n-polyenes ($C_{2n}H_{2n+1}$). Sums for the RASCI(p) and the RASCI(h,p) are the same, as for the RASCI(h) and CASCI calculations. Particle contributions are determinant for the presence of fractional occupancies at RAS3 space.

n	RAS1	RAS2	RAS3
	CASCI		
2	26.0000	4.0000	0.0000
3	38.0000	6.0000	0.0000
4	50.0000	8.0000	0.0000
	RASCI(h)		
2	26.0000	4.0000	0.0000
3	38.0000	6.0000	0.0000
4	50.0000	8.0000	0.0000
	RASCI(p)		
2	26.0000	3.9965	0.0035
3	38.0000	5.9927	0.0073
4	50.0000	7.9889	0.0111

As the dimensions of the system increase, the NOONs profile of the singlet ground state assumes a likeness to the momentum distribution from HEG (see Fig.S1). However, occupations at the overall distribution of occupations is sensible to the size of the basis set (See. Tables S1). While the smallest cc-pVDZ basis introduces some unbalanced distribution among the three orbital spaces, the use of larger basis sets seems to overcome this deficiency. In all cases, in going from

the cc-pVTZ to the cc-pVQZ, occupations at RAS3 increase. Indeed, the introduction of particle contributions into the RASCI wave function is the driving force behind the emergence of non-zero occupancies in RAS3. This phenomenon is evident in Table S2. Notably, both RASCI(h) and CASCI methods fail to allocate any occupation to NO beyond the boundaries of RAS1 and RAS2.

2 Numerical results for atomic series

2.1 Complementary data for Atomic series

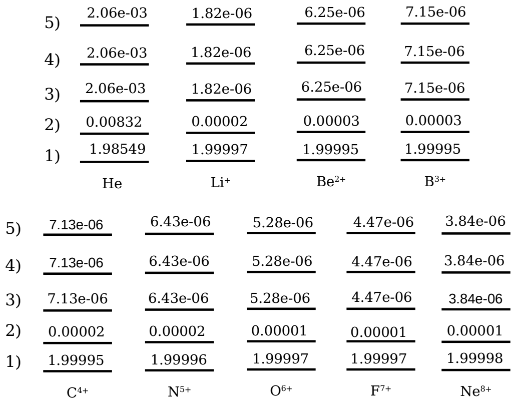

Figure S2: RASCI(h,p)/cc-pVDZ natural orbital occupation numbers (NOONs) representation for the isoelectronic He-series. Each horizontal line represents a natural orbital from the active space (RAS2(2e, 5o)), the numerical value of the respective NOON is placed above. Reported calculations where performed within this selection of the active space.

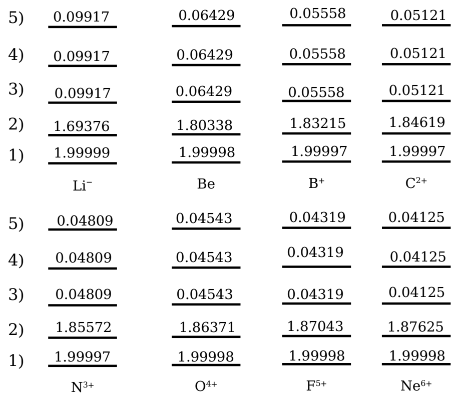

Figure S3: Full valence RASCI(h,p)/cc-pVDZ NOONs representation for the isoelectronic Be-series. Each horizontal line represents a NO from the active space (RAS2(4e, 5o)), the numerical value of the respective NOON is placed above.

In the following, a small change in notation is used: the labels 1S, 2S (2G). and 3S (3G) are used to designate, respectively, the use of a single NOON and cutting the NO space as in the original proposal by Savin; by using a single NOON and taking the sum of the rest of smaller occupations into the DFA with Savins's form of $n^{+}(r_s)$ (or Gori-Giorgi), or by using the sum of occupations criteria as described in the main text (again with the respective form of n^{+}, S or G).

2.2 Performance of different variants of the method

Exact correlation energies (in mhartrees) computed according to the definition of Wigner and Löwdin[S2,S3] for the He- and Be-series and the respective percentage of errors obtained with the different versions of the method (and using the *so*VWN(S) functional form) are depicted in Table S3.

In this case the two methods 2S and 3S give the same results. This is because the sum or occupations has a small value with essentially no differences in the two versions.

Table S3: [a]The 'exact' values of the correlation energies (E_c^{Exact}) were taken from Reference S4, and correspond to the difference between corrected experimental values and Hartree-Fock energies. A RAS2(2e, 5o) and RAS2(4e,5o) active spaces were used for the He- and Be-series respectively. An occupation threshold of 0.001 was chosen.

He-Series		%error (cc-pVTZ)			
Atom	$E_c^{Exact,a}$	RASCI	1S	2S	3S
He	-42.0	-30.64	0.42	-11.04	-11.04
Li^+	-43.5	-97.12	-48.70	-64.31	-64.31
Be^{2+}	-44.3	-96.55	-46.82	-63.63	-63.63
B^{3+}	-44.7	-94.82	-39.46	-59.46	-48.74
C^{4+}	-45.1	-93.72	-36.64	-58.32	-58.32
N^{5+}	-45.3	-92.92	-34.84	-58.06	-58.06
O^{6+}	-45.5	-92.47	-33.87	-58.71	-58.71
F^{7+}	-45.6	-92.11	-33.27	-46.99	-46.999
Ne^{8+}	-45.7	-91.80	-32.88	-60.01	-60.01
Be-Series		%error (cc-pVTZ)			
Atom	E_c^{Exact}	RASCI	1S	2S	3S
Li^-	-72.7	-70.13	-59.62	-63.57	-63.57
Be	-94.3	-60.19	-46.20	-49.65	-49.65
B^+	-111.4	-48.86	-3.91	-10.48	-10.48
C^{2+}	-126.4	-43.01	-18.95	-23.18	-23.18
N^{3+}	-140.5	-39.50	-21.43	-25.58	-25.58
O^{4+}	-154.1	-37.24	-20.92	-25.83	-25.83
F^{5+}	-167.3	-35.23	-20.12	-25.20	-25.20
Ne^{6+}	-180.4	-33.49	-19.38	-24.48	-24.48

2.3 Comparison of correlation energies computed for the Be-series.

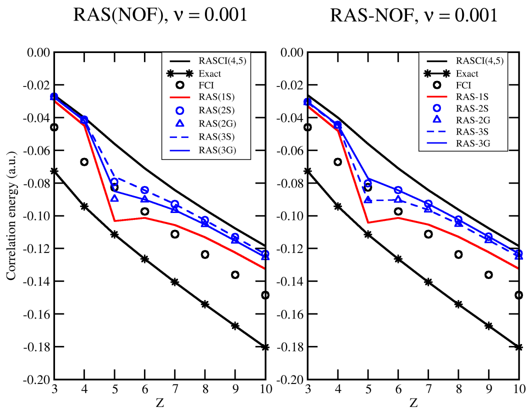

Figure S4: Correlation energies computed for the Be-Series with: *on-top* RAS(NOF) and RAS-NOF schemes. NOONs ($\nu_{threshold} = 0.001$) was chosen in order to get a comparison with the results reported with $\nu_{threshold} = 0.01$. Computed energies are close to the FCI energy than the previous case.

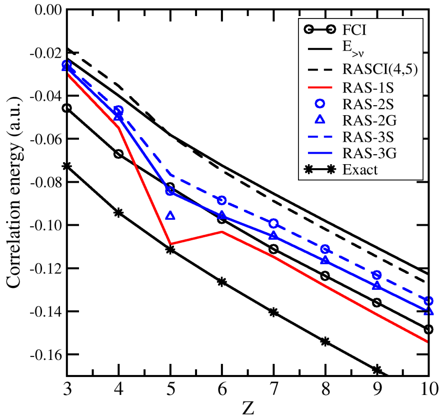

Figure S5: Correlation energies computed for the Be-Series within the RAS-NOF scheme $\nu_{threshold} = 0.01$. The use of a large basis set improves the calculation, however a similar overestimation as that obtained with the cc-pVDZ basis set remains.

2.4 Total energies computed with the cc-pVQZ basis set

Table S4: Total energies (in a.u.) for the He-series computed within different approaches as discussed in main text (with the *so*VWN(G) functional and $\nu = 0.001$), the cc-pVQZ basis set was used.

Atom	HF	RASCI(h,p)	CASSCF	FCI	E>nu	*so*DFT	RAS-*so*DFT
He	-2.8615	-2.8864	-2.8974	-2.9024	-2.8931	-0.0140	-2.9072
Li^{+}	-7.2364	-7.2378	-7.2525	-7.2525	-7.2498	0.0000	-7.2498
Be^{+2}	-13.6113	-13.6143	-13.6302	-13.6304	-13.6264	0.0000	-13.6264
B^{+3}	-21.9862	-21.9904	-22.0054	-22.0054	-22.0017	0.0000	-22.0017
C^{+4}	-32.3611	-32.3661	-32.3811	-32.3812	-32.3736	0.0000	-32.3736
N^{+5}	-44.7360	-44.7417	-44.7565	-44.7566	-44.7492	0.0000	-44.7492
O^{+6}	-59.1108	-59.1169	-59.1312	-59.1313	-59.1242	0.0000	-59.1242
F^{+7}	-75.4857	-75.4921	-75.5060	-75.5061	-75.4993	0.0000	-75.4993
Ne^{+8}	-93.8605	-93.8671	-93.8809	-93.8810	-93.8743	0.0000	-93.8743

Table S5: Total energies (in a.u.) for the He-series computed within different approaches as discussed in main text (with the *so*VWN(S) functional and $\nu = 0.001$), the cc-pVQZ basis set was used.

Atom	HF	RASCI(h,p)	CASSCF	FCI	E>nu	*so*DFT	RAS-*so*DFT
He	-2.8615	-2.8864	-2.8974	-2.9024	-2.8931	-0.0058	-2.8989
Li^{+}	-7.2364	-7.2378	-7.2525	-7.2525	-7.2498	0.0000	-7.2498
Be^{+2}	-13.6113	-13.6143	-13.6302	-13.6304	-13.6264	0.0000	-13.6264
B^{+3}	-21.9862	-21.9904	-22.0054	-22.0054	-22.0017	0.0000	-22.0017
C^{+4}	-32.3611	-32.3661	-32.3811	-32.3812	-32.3736	0.0000	-32.3736
N^{+5}	-44.7360	-44.7417	-44.7565	-44.7566	-44.7492	0.0000	-44.7492
O^{+6}	-59.1108	-59.1169	-59.1312	-59.1313	-59.1242	0.0000	-59.1242
F^{+7}	-75.4857	-75.4921	-75.5060	-75.5061	-75.4993	0.0000	-75.4993
Ne^{+8}	-93.8605	-93.8671	-93.8809	-93.8810	-93.8743	0.0000	-93.8743

Table S6: Total energies (in a.u.) for the Be-series computed within different approaches as discussed in main text (with the *so*VWN(G) functional and $\nu = 0.001$), the cc-pVQZ basis set was used.

Atom	HF	RASCI(h,p)	CASSCF	FCI	E>nu	*so*DFT	RAS-*so*DFT
Li^{-}	-7.4248	-7.4429	-7.4515	-7.47073	-7.4477	-0.0684	-7.5160
Be	-14.5730	-14.6084	-14.6168	-14.64012	-14.6130	-0.0647	-14.6776
B^{+}	-24.2375	-24.2960	-24.2962	-24.31998	-24.2957	-0.0378	-24.3335
C^{+2}	-36.4083	-36.4830	-36.4807	-36.50565	-36.4806	-0.0235	-36.5041
N^{+3}	-51.0819	-51.1707	-51.1673	-51.19304	-51.1673	-0.0155	-51.1828
O^{+4}	-68.2569	-68.3589	-68.3550	-68.38053	-68.3550	-0.0165	-68.3715
F^{+5}	-87.9328	-88.0476	-88.0444	-88.0689	-88.0433	0.0000	-88.0433
Ne^{+6}	-110.1092	-110.2366	-110.2320	-110.25768	-110.2320	0.0000	-110.2320

Table S7: Total energies (in a.u.) for the Be-series computed within different approaches as discussed in main text (with the *so*VWN(S) functional and $\nu = 0.001$), the cc-pVQZ basis set was used.

Atom	HF	RASCI(h,p)	CASSCF	FCI	E>nu	*so*DFT	RAS-*so*DFT
Li^{-}	-7.4248	-7.4429	-7.4515	-7.4707	-7.4477	-0.0460	-7.4937
Be	-14.5730	-14.6084	-14.6168	-14.6401	-14.6130	-0.0441	-14.6571
B^{+}	-24.2375	-24.2960	-24.2962	-24.3200	-24.2957	-0.0261	-24.3218
C^{+2}	-36.4083	-36.4830	-36.4807	-36.5057	-36.4806	-0.0164	-36.4970
N^{+3}	-51.0819	-51.1707	-51.1673	-51.1930	-51.1673	-0.0109	-51.1781
O^{+4}	-68.2569	-68.3589	-68.3550	-68.3805	-68.3550	-0.0117	-68.3666
F^{+5}	-87.9328	-88.0476	-88.0444	-88.0689	-88.0433	0.0000	-88.0433
Ne^{+6}	-110.1092	-110.2366	-110.2320	-110.2577	-110.2320	0.0000	-110.2320

3 H_2 bond dissociation

3.1 Complementary data for H_2 dissociation

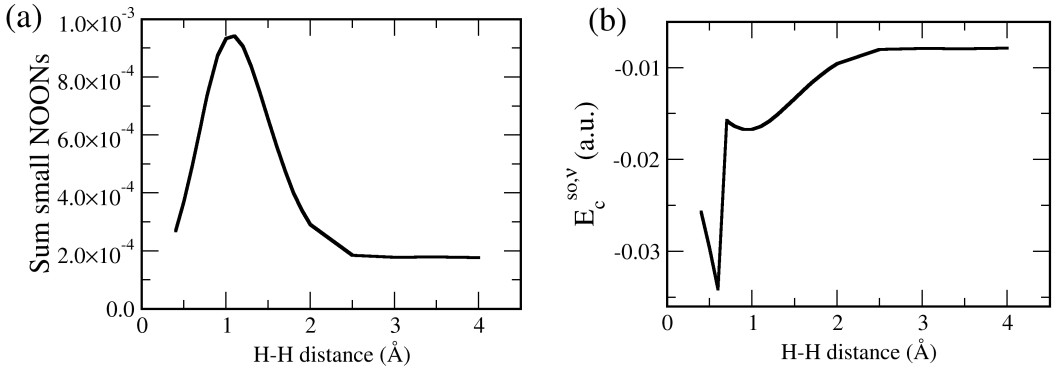

Figure S6: Complete data for (a) sum of small NOONs, and (b) *so*VWN energy (in a.u.) along the H_2 dissociation.

3.2 Potential energy surfaces for H_2

Potential energy surfaces for hydrogen within the 2S and 2G versions are depicted in Fig. S7. Results obtained by addition of the *so*DFT correlation energy to the pristine WFA energy behave similar to those in the RAS-*so*DFT scheme, improving the energy at bonding regions while converging to the WFT solution at long range.

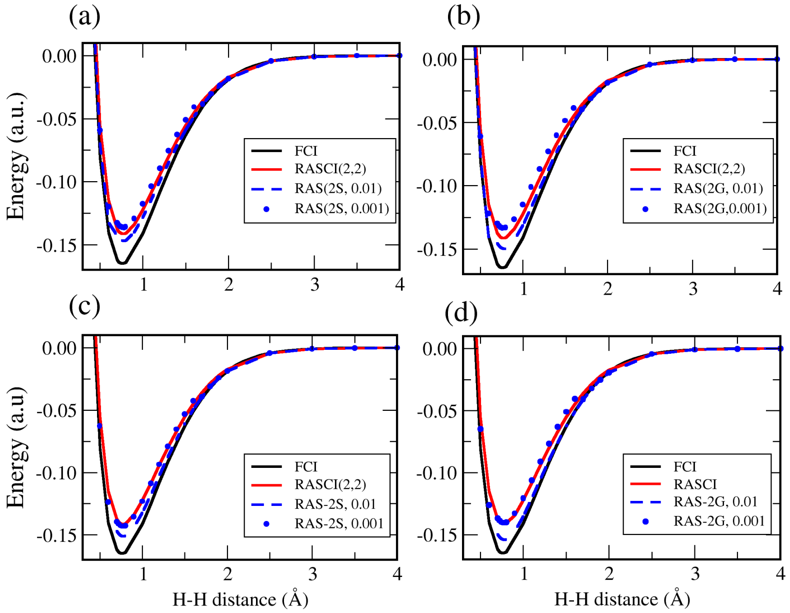

Figure S7: H_2 PES comparison among the $on-top$ (a and b) and the NO splitting (c and d) schemes, threshold values are indicated in the labels.

Table S8: Corresponding total energies for the H_2 PES computed in Figure S7 within the cc-pVDZ basis set in the *on-top* approach. The threshold value is indicated inside squared brackets below the RAS-*so*VWN functional labels.

Distance (Å)	RASCI (2e,2o)	*so*VWN(S) [0.01]	*so*VWN(S) [0.001]	*so*VWN(G) [0.01]	*so*VWN(G) [0.001]
0.4000	-0.9177	-0.9347	-0.9260	-0.9434	-0.9303
0.5000	-1.0535	-1.0731	-1.0626	-1.0831	-1.0672
0.6000	-1.1135	-1.1359	-1.1232	-1.1476	-1.1283
0.7000	-1.1360	-1.1463	-1.1360	-1.1517	-1.1360
0.7200	-1.1378	-1.1482	-1.1378	-1.1537	-1.1379
0.7400	-1.1390	-1.1495	-1.1390	-1.1550	-1.1390
0.7600	-1.1395	-1.1501	-1.1396	-1.1557	-1.1396
0.7800	-1.1396	-1.1503	-1.1397	-1.1559	-1.1397
0.8000	-1.1393	-1.1500	-1.1393	-1.1556	-1.1393
0.9000	-1.1325	-1.1435	-1.1326	-1.1492	-1.1327
1.0000	-1.1208	-1.1317	-1.1210	-1.1375	-1.1210
1.1000	-1.1069	-1.1176	-1.1071	-1.1233	-1.1073
1.2000	-1.0925	-1.1027	-1.0928	-1.1083	-1.0930
1.3000	-1.0784	-1.0882	-1.0790	-1.0935	-1.0792
1.4000	-1.0654	-1.0746	-1.0661	-1.0796	-1.0664
1.5000	-1.0536	-1.0622	-1.0545	-1.0669	-1.0550
1.6000	-1.0432	-1.0513	-1.0443	-1.0557	-1.0449
1.7000	-1.0343	-1.0418	-1.0418	-1.0459	-1.0459
1.8000	-1.0268	-1.0338	-1.0338	-1.0376	-1.0376
1.9000	-1.0206	-1.0272	-1.0272	-1.0307	-1.0307
2.0000	-1.0156	-1.0217	-1.0217	-1.0251	-1.0251
2.5000	-1.0026	-1.0078	-1.0078	-1.0106	-1.0106
3.0000	-0.9993	-1.0044	-1.0044	-1.0072	-1.0072
3.5000	-0.9985	-1.0036	-1.0036	-1.0064	-1.0064
4.0000	-0.9984	-1.0035	-1.0035	-1.0062	-1.0062

Table S9: Corresponding total energies for the H_2 PES computed in Figure S7 within the cc-pVDZ basis set in the RAS-*so*DFT approach. The threshold value is indicated inside squared brackets below the RAS-*so*VWN functional labels.

Distance (Å)	RASCI (2e,2o)	*so*VWN(S) [0.01]	*so*VWN(S) [0.001]	*so*VWN(G) [0.01]	*so*VWN(G) [0.001]
0.4000	-0.9177	-0.9498	-0.9296	-0.9498	-0.9338
0.5000	-1.0535	-1.0900	-1.0665	-1.0900	-1.0712
0.6000	-1.1135	-1.1548	-1.1275	-1.1548	-1.1325
0.7000	-1.1360	-1.1562	-1.1433	-1.1562	-1.1433
0.7200	-1.1378	-1.1582	-1.1451	-1.1582	-1.1452
0.7400	-1.1390	-1.1595	-1.1463	-1.1595	-1.1463
0.7600	-1.1395	-1.1603	-1.1468	-1.1603	-1.1469
0.7800	-1.1396	-1.1605	-1.1469	-1.1605	-1.1469
0.8000	-1.1393	-1.1602	-1.1465	-1.1602	-1.1465
0.9000	-1.1325	-1.1537	-1.1393	-1.1537	-1.1394
1.0000	-1.1208	-1.1417	-1.1270	-1.1417	-1.1271
1.1000	-1.1069	-1.1271	-1.1125	-1.1271	-1.1126
1.2000	-1.0925	-1.1117	-1.0974	-1.1117	-1.0976
1.3000	-1.0784	-1.0964	-1.0827	-1.0964	-1.0830
1.4000	-1.0654	-1.0821	-1.0692	-1.0821	-1.0695
1.5000	-1.0536	-1.0690	-1.0570	-1.0690	-1.0575
1.6000	-1.0432	-1.0574	-1.0463	-1.0574	-1.0469
1.7000	-1.0343	-1.0473	-1.0432	-1.0473	-1.0473
1.8000	-1.0268	-1.0387	-1.0349	-1.0387	-1.0387
1.9000	-1.0206	-1.0316	-1.0280	-1.0316	-1.0316
2.0000	-1.0156	-1.0258	-1.0224	-1.0258	-1.0258
2.5000	-1.0026	-1.0109	-1.0081	-1.0109	-1.0109
3.0000	-0.9993	-1.0074	-1.0046	-1.0074	-1.0074
3.5000	-0.9985	-1.0067	-1.0039	-1.0067	-1.0067
4.0000	-0.9984	-1.0065	-1.0037	-1.0065	-1.0065

3.3 Energy dependence on ν parameter

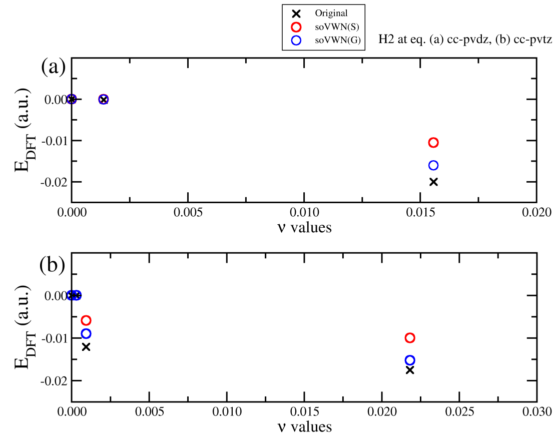

Figure S8: DFT energy dependence on the parameter *nu* for the H_2 ground state at $r_{HH} = 0.74$ Å computed with the (a) cc-pVDZ and (b) cc-pVTZ basis sets.

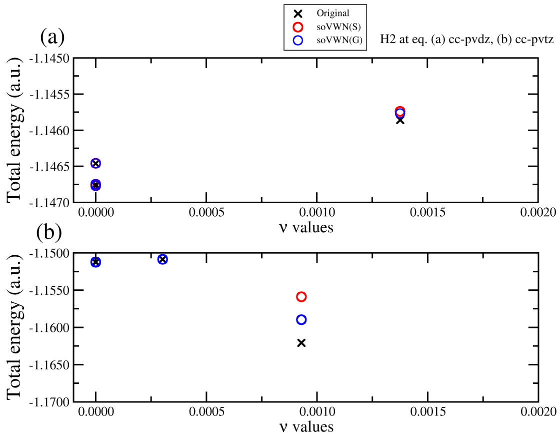

Figure S9: Total energy dependence on the parameter *nu* for the H_2 ground state at $r_{HH} = 0.74$ Å computed with the (a) cc-pVDZ and (b) cc-pVTZ basis sets.

3.4 Basis set dependence

Table S10: Dissociation energies (in mHatree) for H_2 computed with different basis sets. Threshold for the sum of occupations used is 0.001. Experimental value is 164.5 mHartree.[S5]

basis	RASCI	$\mathscr{E}_0^{\mathrm{WFA},\nu}$	*so*VWN(G)	FCI
cc-pVDZ	141.3	148.5	162.2	163.9
cc-pVTZ	150.5	159.3	160.2	171.1
cc-pVQZ	150.7	159.8	160.8	172.2

4 C_2H_4 molecular torsion

4.1 Complementary data for C_2H_4 torsion

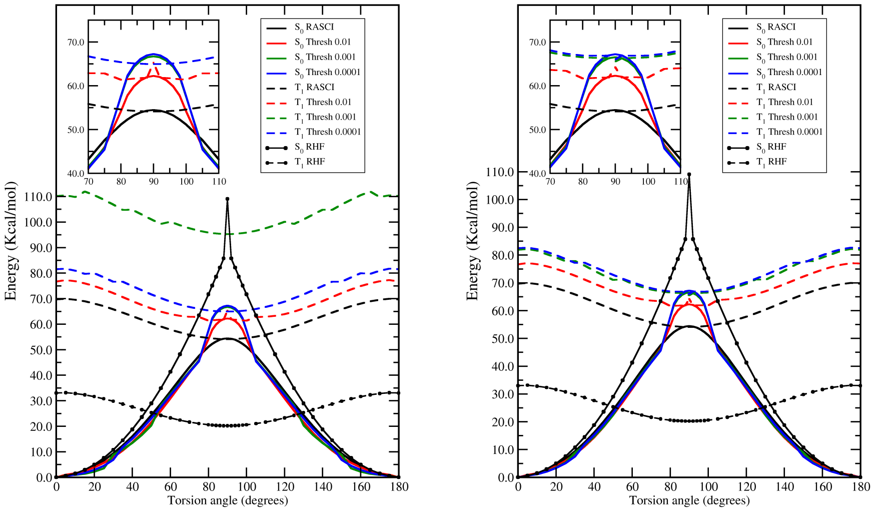

Figure S10: Ethylene potential energy surfaces for the singlet ground state and the lowest lying triplet state. Left: RAS-*so*DFT profiles obtained with the Savin (left) and Gori (right) $n_+(r_s)$ expressions, and with different values for the sum of occupations threshold.

4.2 Potential energy surfaces for C_2H_4

In the following tables, data for twisting modes of ethylene is summarized, values correspond to those for the PES shown above in Figure S10. Threshold values are indicated below method labels.

Table S11: PES data for the ethylene singlet state torsion corresponding values from Figure S10

			RAS-*so*VWN(S)			RAS-*so*VWN(G)		
Angle (degrees)	RHF	RASCI	0.01	0.001	0.0001	0.01	0.001	0.0001
0	0.0000	0.0000	0.0000	0.0000	0.0000	0.0000	0.0000	0.0000
5	0.6004	0.6965	0.8785	0.6212	0.5585	1.0103	0.6338	0.5334
10	1.4736	1.4495	1.5123	1.1609	1.0793	1.5876	0.8722	0.9726
15	2.9272	2.6983	2.5540	2.0708	2.0080	2.5414	1.3240	1.7633
20	4.9585	4.4365	4.0286	3.5078	3.3509	3.8968	2.1712	2.9493
25	7.5639	6.6579	5.9362	5.3276	5.0640	5.6601	3.4952	4.5118
30	10.7385	9.3436	8.2831	8.8479	7.4987	7.8627	8.8165	6.8524
35	14.4762	12.4812	11.0881	11.5085	11.8411	10.5171	10.8936	11.8411
40	18.7696	16.0454	14.3511	14.6335	15.1732	13.6421	13.4664	15.1167
45	23.6098	19.9987	18.1225	18.2731	18.9822	17.3067	16.6290	18.8880
50	28.9866	24.2972	22.3895	22.3833	23.2178	21.4859	20.3313	23.0861
55	34.8878	28.8717	27.0143	28.1438	27.7108	26.0730	28.1877	27.5477
60	41.2995	33.6471	31.8398	32.7121	32.2916	30.9111	32.5489	32.0971
65	48.2056	38.4852	36.8160	37.3243	36.8850	35.9500	37.0293	36.6591
70	55.5877	43.2040	41.7733	41.7984	41.3842	41.0391	41.4282	41.1270
75	63.4249	47.5464	46.4796	46.0843	45.7078	45.9274	45.7956	45.4568
80	71.6932	51.1546	54.4929	57.6430	57.8124	54.1666	57.5301	57.6242
82	75.1152	52.2966	58.0321	62.0732	62.4372	57.7936	62.0481	62.2866
84	78.5999	53.2191	60.0464	64.2632	64.7527	59.8895	64.3323	64.6586
86	82.1454	53.8968	61.3265	65.5810	66.1583	61.2324	65.7316	66.1207
88	85.7464	54.3172	62.0419	66.2964	66.9553	61.9917	66.4846	66.9490
90	109.0452	54.4605	62.2866	66.5223	67.2251	62.2427	66.7105	67.2376
92	85.7464	54.3172	62.0419	66.2964	66.9553	61.9917	66.4846	66.9490
94	82.1454	53.8968	61.3265	65.5810	66.1583	61.2324	65.7316	66.1207
96	78.5999	53.2191	60.0464	64.2632	64.7527	59.8895	64.3323	64.6586
98	75.1152	52.2966	58.0321	62.0732	62.4372	57.7936	62.0481	62.2866
100	71.6932	51.1546	54.4929	57.6430	57.8124	54.1666	57.5301	57.6242
105	63.4249	47.5464	46.4796	46.0843	45.7078	45.9274	45.7956	45.4568
110	55.5877	43.2040	41.7733	41.7984	41.3842	41.0391	41.4282	41.1270
115	48.2056	38.4852	36.8160	37.3243	36.8850	35.9500	37.0293	36.6591
120	41.2995	33.6471	31.8398	32.7121	32.2916	30.9111	32.5489	32.0971
125	34.8878	28.8717	27.0143	28.1438	27.7108	26.0730	28.1877	27.5477
130	28.9866	24.2972	22.3895	22.3833	23.2178	21.4859	20.3313	23.0861
135	23.6098	19.9987	18.1225	18.2731	18.9822	17.3067	16.6290	18.8880
140	18.7696	16.0454	14.3511	14.6335	15.1732	13.6421	13.4664	15.1167
145	14.4762	12.4812	11.0881	11.5085	11.8411	10.5171	10.8936	11.8411
150	10.7385	9.3436	8.2831	8.8479	7.4987	7.8627	8.8165	6.8524
155	7.5639	6.6579	5.9362	5.3276	5.0640	5.6601	3.4952	4.5118
160	4.9585	4.4365	4.0286	3.5078	3.3509	3.8968	2.1712	2.9493
165	2.9272	2.6983	2.5540	2.0708	2.0080	2.5414	1.3240	1.7633
170	1.4736	1.4495	1.5123	1.1609	1.0793	1.5876	0.8722	0.9726
175	0.6004	0.6965	0.8785	0.6212	0.5585	1.0103	0.6338	0.5334
180	0.0000	0.0000	0.0000	0.0000	0.0000	0.0000	0.0000	0.0000

Table S12: PES data for the ethylene triplet state torsion corresponding values from Figure S10

			RAS-*so*VWN(S)			RAS-*so*VWN(G)		
Angle (degrees)	RHF	RASCI	0.01	0.001	0.0001	0.01	0.001	0.0001
0	33.0100	69.6912	76.6754	81.9025	82.4736	76.6754	110.1781	81.5511
5	33.1438	69.9610	77.1711	82.1724	82.6932	77.3029	110.4605	81.7205
10	32.8258	69.5657	76.7256	81.5950	82.1033	76.8009	109.7828	80.9111
15	32.3094	68.9319	76.0039	80.3338	81.1997	75.9914	111.9414	79.7188
20	31.6138	68.0722	75.0438	79.1227	79.9447	74.9121	110.5170	79.9823
25	30.7645	67.0306	73.8830	77.7798	78.5893	73.6069	108.7850	78.3194
30	29.7915	65.8320	72.5777	77.8739	77.1962	72.1573	106.8397	76.6126
35	28.7285	64.5268	71.1784	76.2173	77.2715	70.6074	104.7564	76.6503
40	27.6105	63.1525	69.7539	74.3724	75.7780	69.0449	104.8505	74.9434
45	26.4732	61.7532	68.3546	72.6970	74.3097	67.5388	102.9053	73.2805
50	25.3514	60.3852	67.0431	71.0905	72.8727	66.1395	101.0165	71.6741
55	24.2779	59.0675	65.8697	71.2474	71.4984	64.9284	99.2594	70.1807
60	23.2830	57.8564	64.8719	69.7100	70.2120	63.9432	100.1066	68.8315
65	22.3938	56.7708	64.1126	68.5554	69.0637	63.2467	98.7261	67.6832
70	21.6337	55.8421	63.6106	67.5953	68.0848	62.8764	97.5463	66.7231
75	21.0222	55.1016	63.3910	66.9176	67.3380	62.8388	96.5800	65.9512
80	20.5744	54.5557	61.6779	66.5034	66.9239	61.3516	95.8709	65.3928
82	20.4437	54.3988	61.7218	66.4093	66.8674	61.4834	95.6638	65.2296
84	20.3415	54.2733	61.7908	66.3528	66.8549	61.6340	95.5007	65.1041
86	20.2683	54.1854	61.8473	66.3215	66.8800	61.7532	95.3689	65.0225
88	20.2212	54.1290	61.8787	66.2901	66.9051	61.8285	95.2873	64.9598
90	20.2119	54.1164	64.2381	65.5245	65.9952	65.2735	95.2622	64.9472
92	20.2212	54.1290	61.8787	66.2901	66.9051	61.8285	95.2873	64.9598
94	20.2683	54.1854	61.9289	66.3215	66.8800	61.7532	95.3689	65.0225
96	20.3415	54.2733	61.9289	66.3528	66.8549	61.6340	95.5007	65.1041
98	20.4437	54.3988	61.8975	66.4093	66.8674	61.4834	95.6638	65.2296
100	20.5744	54.5557	61.8662	66.5034	66.9239	61.3516	95.8709	65.3928
105	21.0222	55.1016	63.8616	66.9176	67.3380	62.8388	96.5800	65.9512
110	21.6337	55.8421	64.0122	67.5953	68.0848	62.8764	97.5463	66.7231
115	22.3938	56.7708	64.3825	68.5554	69.0637	63.2467	98.7261	67.6832
120	23.2830	57.8564	65.0162	69.7100	70.2120	63.9432	100.1066	68.8315
125	24.2779	59.0675	65.8822	71.2474	71.4984	64.9284	99.2594	70.1807
130	25.3514	60.3852	66.9615	71.0905	72.8727	66.1395	101.0165	71.6741
135	26.4732	61.7532	68.1789	72.6970	74.3097	67.5388	102.9053	73.2805
140	27.6105	63.1525	69.5092	74.3724	75.7780	69.0449	104.8505	74.9434
145	28.7285	64.5268	70.8835	76.2173	77.2715	70.6074	104.7564	76.6503
150	29.7915	65.8320	72.2640	77.8739	77.1962	72.1573	106.8397	76.6126
155	30.7645	67.0306	73.5629	77.7798	78.5893	73.6069	108.7850	78.3194
160	31.6138	68.0722	74.7426	79.1227	79.9447	74.9121	110.5170	79.9823
165	32.3094	68.9319	75.7467	80.3338	81.1997	75.9914	111.9414	79.7188
170	32.8258	69.5657	76.5248	81.5950	82.1033	76.8009	109.7828	80.9111
175	33.1438	69.9610	77.0519	82.1724	82.6932	77.3029	110.4605	81.7205
180	33.0100	69.6912	76.9640	81.9025	82.4736	76.6754	110.1781	81.5511

4.3 Energy dependence on ν parameter

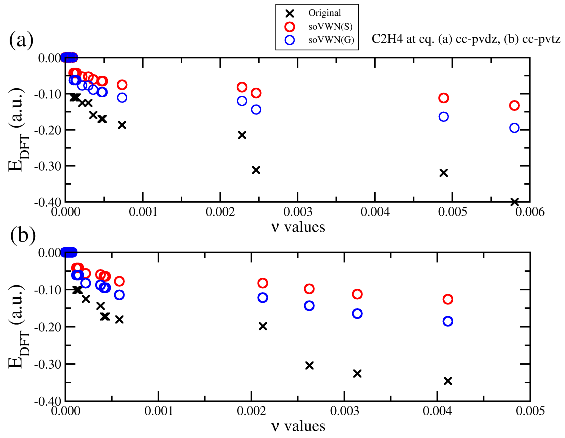

Figure S11: DFT energy dependence on the parameter *nu* for the C_2H_4 ground state (optimized at the CCSD(T)/cc-pVDZ level) computed with the (a) cc-pVDZ and (b) cc-pVTZ basis sets.

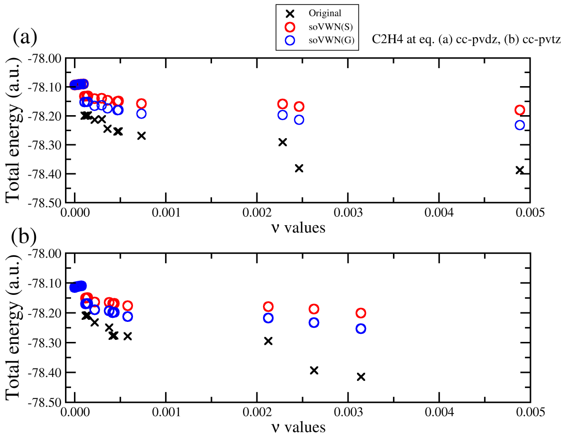

Figure S12: Total energy dependence on the parameter *nu* for the C_2H_4 ground state (optimized at the CCSD(T)/cc-pVDZ level) computed with the (a) cc-pVDZ and (b) cc-pVTZ basis sets.

5 N_2 triple bond dissociation

5.1 RASCI Natural orbital occupations computed for the N_2

In order to try to understand the overestimations made by the density functional in the region of dissociation, corresponding natural orbital occupations are plotted in Figures S14 and S15. From these it is observed that as the number of effective unpaired electrons increase, more natural orbitals acquire occupations that are not negligible and that are close to the chosen threshold. In the case of RAS(NOF0,2) the situation is not compensated by the sum of occupations. The problem arise due to the use of a single value for the occupation threshold. In fact this is the major problem with Savin's proposal, as the distribution of occupations from Figures S14 and S15 suggest, NO occupations can change along the PES. Furthermore, as the HG index shows (see Fig. S13), at dissociation it is expected a total amount of six unpaired electrons, during the transition, some occupations will achieve a value of 1.0 and the distribution profile will acquire a kind of "step" (see distribution of NO occupations at 2.5 Å of separation in Figure S14). At these long-range atomic separations, NO occupations in the RAS2 space tend to be close to the unity, and the density functional energy contribution converges to zero.

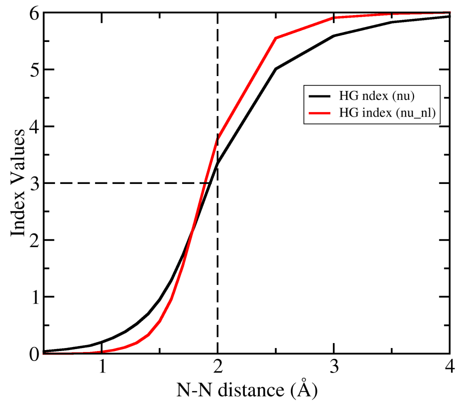

Figure S13: Computed values for the effective unpaired electrons accounted from the Head-Gordon "nu" and "nu_nl" indexes along the dissociation of N_2.

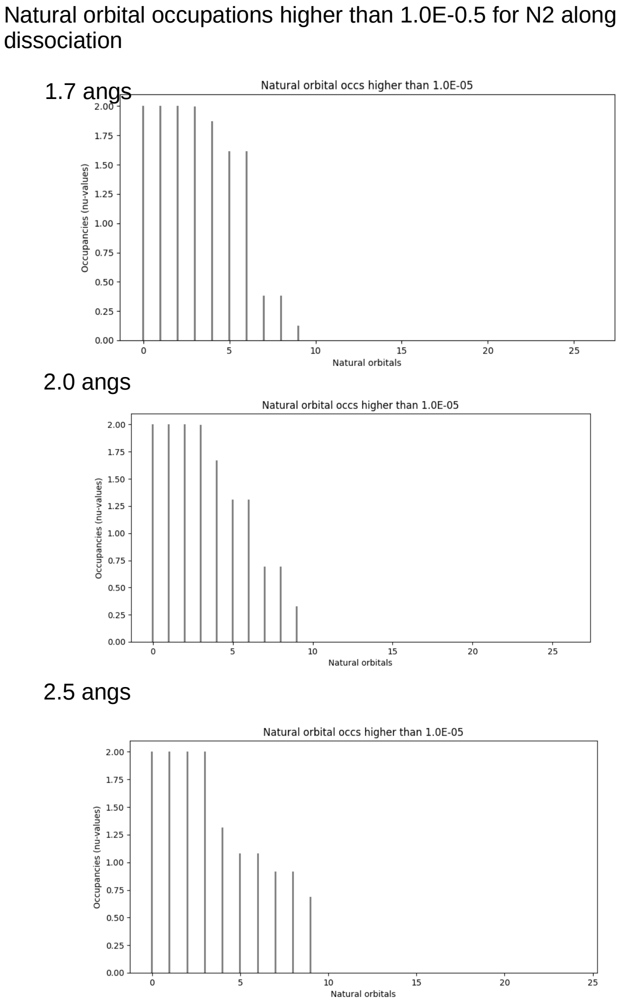

Figure S14: Natural orbital occupations (higher than a 1.0E-05) at different separations.

5.2 Potential energy surfaces for N_2

In Fig. S16, the potential energy surfaces computed within the 1S, single NOON scheme are depicted for different values used for the threshold parameter (ν), it is particularly interesting the effect of using a large threshold since results are largely affected giving wrong PES, while the proper NO space splitting seems to alleviate the problem, in bot cases the best choice resembles in the consideration and use of small NOONs. In Fig. S17, a bigger comparison is shown involving the rest of schemes.

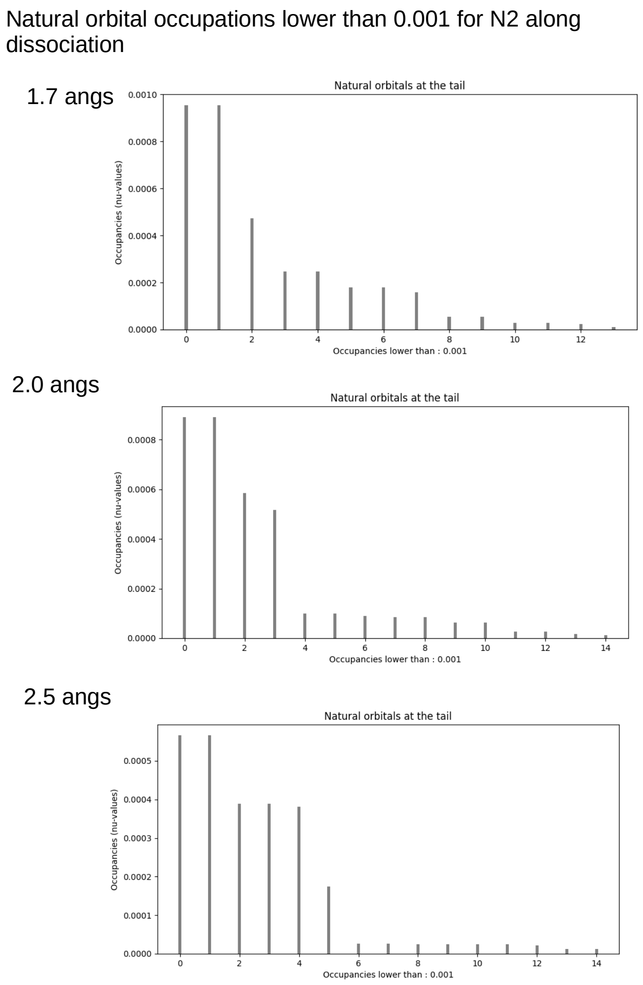

Figure S15: Natural orbital occupations (lower than a threshold of 0.001) at different separations.

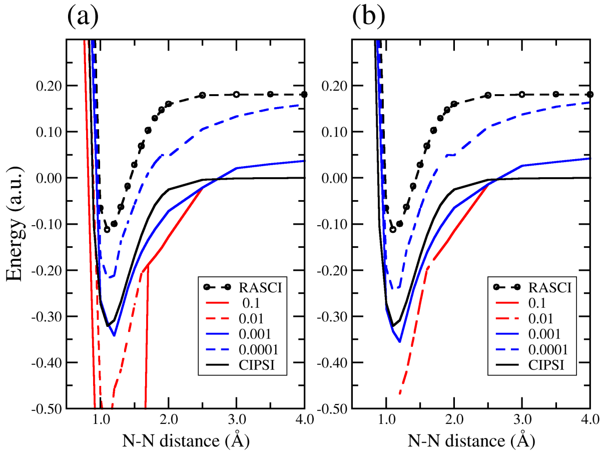

Figure S16: Absolute energy profiles along the N_2 dissociation computed at the RASCI (dashed black) and CIPSI (solid black) computed with the cc-pVDZ basis set and their comparison with (a) *on-top* DFT calculations and (b) splitting the natural orbital space. In both cases the 1S single NOON based threshold scheme was used (number in label resembles the occupation threshold used).

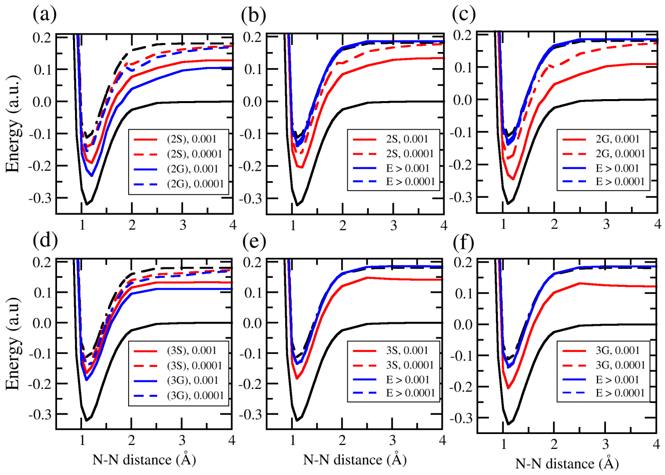

Figure S17: General comparison of N_2 PES computed with the different schemes presented in this work and standard RASCI and CIPSI calculations within the cc-pVDZ basis set. The $E_>$ label indicates such cases where no DFT correlation energy is incorpored into the final energy, i.e. these energies correspond to the WFT energy computed in the space of NOs with large occupations.

Table S13: Correlation energies (computed with *so*VWN(S) functional) and E_v values computed within the cc-pVDZ basis set, different values of the occupation threshold are indicated. All RAS3 data corresponds to the calculation made by cutting the NO space at the end of RAS2 and ussing all occupations in RAS3. All data is in atomic units, E_c-DFT is the correlation energy delivered by the density functional and $E_{>v}$ is the WFT energy computed with natural orbitals with occupation numbers larger than threshold.

	Threshold values $E_{>v}$				Threshold values E_c-DFT			
d (Å)	0.1	0.01	0.001	0.0001	0.1	0.01	0.001	0.0001
0.5	0.0000	0.0000	-103.0837	-103.0876	-0.1493	-0.1022	-0.0425	0.0000
0.7	0.0000	-107.6549	-107.6852	-107.6890	-0.1936	-0.1193	-0.0447	0.0000
1.0	0.0000	-109.0378	-109.0486	-109.0521	-0.1321	-0.0915	-0.0431	0.0000
1.1	0.0000	-109.0849	-109.0950	-109.0997	-0.1446	-0.0913	-0.0484	0.0000
1.2	0.0000	-109.0702	-109.0825	-109.0852	-0.1120	-0.0913	-0.0400	0.0000
1.3	0.0000	-109.0282	-109.0406	-109.0431	-0.1119	-0.0923	-0.0431	0.0000
1.4	0.0000	-108.9776	-108.9901	-108.9924	-0.1109	-0.0951	-0.0462	0.0000
1.5	0.0000	-108.9275	-108.9415	-108.9425	-0.1111	-0.0994	-0.0420	0.0000
1.6	0.0000	-108.8827	-108.8973	-108.8981	-0.1126	-0.1041	-0.0417	0.0000
1.7	-108.8459	-108.8459	-108.8606	-108.8614	-0.1043	-0.1043	-0.0456	0.0000
1.8	-108.8183	-108.8183	-108.8323	-108.8329	-0.1033	-0.1033	-0.0436	0.0000
1.9	-108.7995	-108.7995	-108.8119	-108.8123	-0.1006	-0.1006	-0.0448	0.0000
2.0	-108.7878	-108.7878	-108.7981	-108.7984	-0.0961	-0.0961	-0.0416	0.0000
2.5	-108.7744	-108.7744	-108.7765	-108.7770	-0.0680	-0.0680	-0.0358	0.0000
3.0	-108.7740	-108.7740	-108.7742	-108.7747	-0.0529	-0.0529	-0.0422	0.0000
3.5	-108.7742	-108.7742	-108.7743	-108.7746	-0.0480	-0.0480	-0.0439	0.0000
4.0	-108.7744	-108.7744	-108.7744	-108.7747	-0.0481	-0.0481	-0.0441	0.0000

Table S14: RAS-*so*VWN(S) total energies and comparison with CASSCF and FCI calculations within the cc-pVDZ basis set, different values of the occupation threshold are indicated. All RAS3 data corresponds to the calculation made by cutting the NO space at the end of RAS2 and ussing all occupations in RAS3. All data is in atomic units, E_c-DFT is the correlation energy delivered by the density functional and $E_{>v}$ is the WFT energy computed with natural orbitals with occupation numbers larger than threshold. Dotted empty values cannot be computed since the threshold values pushes to cut the RAS2 space.

				Threshold values Total RAs-*so*VWN(S) energy			
d (Å)	CIPSI	CASSCF(6,6)	RASCI	0.1	0.01	0.001	0.0001
0.5		0.0000	-103.0837	...	...	-103.1262	-103.0876
0.7	0.0000	-107.6549	-107.6852	...	-107.7741	-107.7300	-107.6890
1.0	0.0000	-109.0378	-109.0486	...	-109.1293	-109.0916	-109.0521
1.1	0.0000	-109.0849	-109.0950	...	-109.1762	-109.1434	-109.0997
1.2	0.0000	-109.0702	-109.0825	...	-109.1616	-109.1225	-109.0852
1.3	0.0000	-109.0282	-109.0406	...	-109.1205	-109.0838	-109.0431
1.4	0.0000	-108.9776	-108.9901	...	-109.0728	-109.0363	-108.9924
1.5	0.0000	-108.9275	-108.9415	...	-109.0269	-108.9834	-108.9425
1.6	0.0000	-108.8827	-108.8973	...	-108.9868	-108.9390	-108.8981
1.7	-108.8459	-108.8459	-108.8606	-108.9502	-108.9502	-108.9061	-108.8614
1.8	-108.8183	-108.8183	-108.8323	-108.9216	-108.9216	-108.8760	-108.8329
1.9	-108.7995	-108.7995	-108.8119	-108.9000	-108.9000	-108.8567	-108.8123
2.0	-108.7878	-108.7878	-108.7981	-108.8838	-108.8838	-108.8397	-108.7984
2.5	-108.7744	-108.7744	-108.7765	-108.8423	-108.8423	-108.8123	-108.7770
3.0	-108.7740	-108.7740	-108.7742	-108.8269	-108.8269	-108.8164	-108.7747
3.5	-108.7742	-108.7742	-108.7743	-108.8223	-108.8223	-108.8182	-108.7746
4.0	-108.7744	-108.7744	-108.7744	-108.8224	-108.8224	-108.8185	-108.7747

Table S15: Correlation energies (computed with *so*VWN(G) functional) and E_v values computed within the cc-pVDZ basis set, different values of the occupation threshold are indicated. All RAS3 data corresponds to the calculation made by cutting the NO space at the end of RAS2 and ussing all occupations in RAS3. All data is in atomic units, E_c-DFT is the correlation energy delivered by the density functional and $E_{>v}$ is the WFT energy computed with natural orbitals with occupation numbers larger than threshold.

	Threshold values $E_{>v}$				Threshold values E_c-DFT			
d (Å)	0.1	0.01	0.001	0.0001	0.1	0.01	0.001	0.0001
0.5	0.0000	0.0000	-103.0837	-103.0876	-0.2147	-0.1470	-0.0611	0.0000
0.7	0.0000	-107.6549	-107.6852	-107.6890	-0.2792	-0.1720	-0.0645	0.0000
1.0	0.0000	-109.0378	-109.0486	-109.0521	-0.1910	-0.1323	-0.0623	0.0000
1.1	0.0000	-109.0849	-109.0950	-109.0997	-0.2093	-0.1321	-0.0700	0.0000
1.2	0.0000	-109.0702	-109.0825	-109.0852	-0.1621	-0.1322	-0.0579	0.0000
1.3	0.0000	-109.0282	-109.0406	-109.0431	-0.1621	-0.1337	-0.0625	0.0000
1.4	0.0000	-108.9776	-108.9901	-108.9924	-0.1607	-0.1378	-0.0669	0.0000
1.5	0.0000	-108.9275	-108.9415	-108.9425	-0.1610	-0.1440	-0.0608	0.0000
1.6	0.0000	-108.8827	-108.8973	-108.8981	-0.1632	-0.1508	-0.0604	0.0000
1.7	-108.8459	-108.8459	-108.8606	-108.8614	-0.1511	-0.1511	-0.0660	0.0000
1.8	-108.8183	-108.8183	-108.8323	-108.8329	-0.1497	-0.1497	-0.0632	0.0000
1.9	-108.7995	-108.7995	-108.8119	-108.8123	-0.1457	-0.1457	-0.0649	0.0000
2.0	-108.7878	-108.7878	-108.7981	-108.7984	-0.1392	-0.1392	-0.0603	0.0000
2.5	-108.7744	-108.7744	-108.7765	-108.7770	-0.0985	-0.0985	-0.0519	0.0000
3.0	-108.7740	-108.7740	-108.7742	-108.7747	-0.0767	-0.0767	-0.0611	0.0000
3.5	-108.7742	-108.7742	-108.7743	-108.7746	-0.0696	-0.0696	-0.0636	0.0000
4.0	-108.7744	-108.7744	-108.7744	-108.7747	-0.0696	-0.0696	-0.0639	0.0000

Table S16: RAS-*so*VWN(G) total energies and comparison with CASSCF and FCI calculations within the cc-pVDZ basis set, different values of the occupation threshold are indicated. All RAS3 data corresponds to the calculation made by cutting the NO space at the end of RAS2 and ussing all occupations in RAS3. All data is in atomic units, E_c-DFT is the correlation energy delivered by the density functional and $E_{>v}$ is the WFT energy computed with natural orbitals with occupation numbers larger than threshold. Dotted empty values cannot be computed since the threshold values pushes to cut the RAS2 space.

				Threshold values<br>Total RAs-*so*VWN(G) energy			
d (Å)	CIPSI	CASSCF(6,6)	RASCI	0.1	0.01	0.001	0.0001
0.5	-103.2506	-103.1412	-103.0698	...	....	-103.1448	-103.0876
0.7	-107.8521	-107.6829	-107.6681	...	-107.8572	-107.7498	-107.6890
1.0	-109.2324	-109.0449	-109.0257	...	-109.1809	-109.1109	-109.0521
1.1	-109.2811	-109.0908	-109.0722	...	-109.2271	-109.1650	-109.0997
1.2	-109.2686	-109.0757	-109.0601	...	-109.2148	-109.1405	-109.0852
1.3	-109.2282	-109.0330	-109.0234	...	-109.1743	-109.1031	-109.0431
1.4	-109.1782	-108.9810	-108.9781	...	-109.1279	-109.0570	-108.9924
1.5	-109.1286	-108.9296	-108.9324	...	-109.0854	-109.0023	-108.9425
1.6	-109.0842	-108.8841	-108.8912	...	-109.0481	-108.9577	-108.8981
1.7	-109.0476	-108.8472	-108.8571	-108.9971	-109.0117	-108.9266	-108.8614
1.8	-109.0192	-108.8197	-108.8311	-108.9680	-108.9820	-108.8955	-108.8329
1.9	-108.9988	-108.8011	-108.8125	-108.9452	-108.9576	-108.8768	-108.8123
2.0	-108.9851	-108.7899	-108.8002	-108.9270	-108.9372	-108.8583	-108.7984
2.5	-108.9640	-108.7777	-108.7813	-108.8729	-108.8750	-108.8284	-108.7770
3.0	-108.9614	-108.7754	-108.7792	-108.8507	-108.8509	-108.8353	-108.7747
3.5	-108.9605	-108.7769	-108.7791	-108.8439	-108.8439	-108.8379	-108.7746
4.0	-108.9603	-108.7770	-108.7793	-108.8440	-108.8441	-108.8383	-108.7747

Table S17: *On-Top* PES total energy results computed within the *so*VWN(S) and *so*VWN(G) functionals, the cc-pVDZ basis set and different threshold values as indicated.

	Threshold values			
	RAS(*so*VWN(S))			
d (Å)	0.1	0.01	0.001	0.0001
0.5	-103.0698	-103.2489	-103.1844	-103.1203
0.7	-107.6681	-107.9017	-107.7960	-107.7192
1.0	-109.0257	-109.2721	-109.1343	-109.0733
1.1	-109.0722	-109.3469	-109.1824	-109.1249
1.2	-109.0601	-109.2243	-109.1721	-109.1060
1.3	-109.0234	-109.2145	-109.1353	-109.0724
1.4	-108.9781	-109.1995	-109.0891	-109.0300
1.5	-108.9324	-109.1851	-109.0435	-108.9806
1.6	-108.8912	-109.1774	-109.0038	-108.9383
1.7	-108.8571	-108.9713	-108.9713	-108.9080
1.8	-108.8311	-108.9452	-108.9452	-108.8789
1.9	-108.8125	-108.9242	-108.9242	-108.8604
2.0	-108.8002	-108.9069	-108.9069	-108.8444
2.5	-108.7813	-108.8556	-108.8556	-108.8277
3.0	-108.7792	-108.8370	-108.8370	-108.8272
3.5	-108.7791	-108.8320	-108.8320	-108.8271
4.0	-108.7793	-108.8315	-108.8315	-108.8273
	Threshold values			
	RAS(*so*VWN(G))			
d (Å)	0.1	0.01	0.001	0.0001
0.5	-103.3274	-103.2347	-103.1425	-103.1025
0.7	-108.0049	-107.8526	-107.7419	-107.7015
1.0	-109.3820	-109.1828	-109.0946	-109.0567
1.1	-109.4697	-109.2317	-109.1485	-109.1015
1.2	-109.2978	-109.2222	-109.1265	-109.0920
1.3	-109.3002	-109.1855	-109.0944	-109.0542
1.4	-109.2987	-109.1388	-109.0533	-109.0059
1.5	-109.2985	-109.0934	-109.0022	-108.9612
1.6	-109.3060	-109.0544	-108.9595	-108.9209
1.7	-109.0225	-109.0225	-108.9308	-108.8876
1.8	-108.9965	-108.9965	-108.9005	-108.8618
1.9	-108.9744	-108.9744	-108.8819	-108.8429
2.0	-108.9549	-108.9549	-108.8642	-108.8295
2.5	-108.8890	-108.8890	-108.8486	-108.8102
3.0	-108.8630	-108.8630	-108.8488	-108.8052
3.5	-108.8557	-108.8557	-108.8487	-108.7956
4.0	-108.8550	-108.8550	-108.8489	-108.7904

Table S18: Selected RAS-*so*DFT results PES total energy results computed within the *so*VWN(S) and *so*VWN(G) functionals (the threshold value was set to 0.001 in both cases), the cc-pVTZ basis set and different threshold values as indicated.

d (Å)	CIPSI	RASCI	CASSCF(6,6)	RAS-*so*VWN(S)	RAS-*so*VWN(G)
0.5	-103.8083	-103.4813	-103.4725	-103.5428	-103.5697
0.7	-108.1109	-107.8040	-107.7960	-107.8623	-107.8881
0.9	-109.2309	-108.9113	-108.8905	-108.9692	-108.9949
1.0	-109.3714	-109.0588	-109.0830	-109.1163	-109.1420
1.1	-109.4004	-109.0964	-109.1194	-109.1552	-109.1816
1.2	-109.3762	-109.0784	-109.1001	-109.1360	-109.1618
1.3	-109.3303	-109.0374	-109.0557	-109.0936	-109.1188
1.4	-109.2790	-108.9922	-109.0030	-109.0459	-109.0700
1.5	-109.2302	-108.9495	-108.9513	-109.0047	-109.0295
1.6	-109.1871	-108.9105	-108.9054	-108.9687	-108.9949
1.7	-109.1508	-108.8772	-108.8679	-108.9355	-108.9617
1.8	-109.1210	-108.8509	-108.8397	-108.9082	-108.9339
1.9	-109.0970	-108.8317	-108.8204	-108.8870	-108.9119
2.0	-109.0779	-108.8185	-108.8084	-108.8741	-108.8990
2.5	-109.0292	-108.7976	-108.7949	-108.8528	-108.8776
3.0	-109.0157	-108.7956	-108.7938	-108.8509	-108.8758
3.5	-109.0121	-108.7956	-108.7946	-108.8332	-108.8501
4.0	-109.0108	-108.7960	-108.7947	-108.8210	-108.8322

5.3 Energy dependence on ν parameter

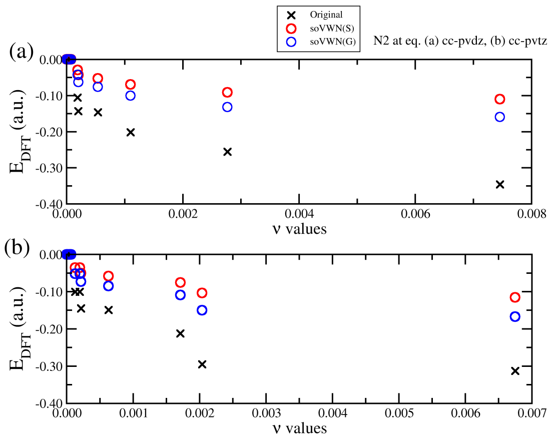

Figure S18: DFT energy dependence on the parameter *nu* for the N_2 ground state at $r_{NN} = 1.10$ Å computed with the (a) cc-pVDZ and (b) cc-pVTZ basis sets.

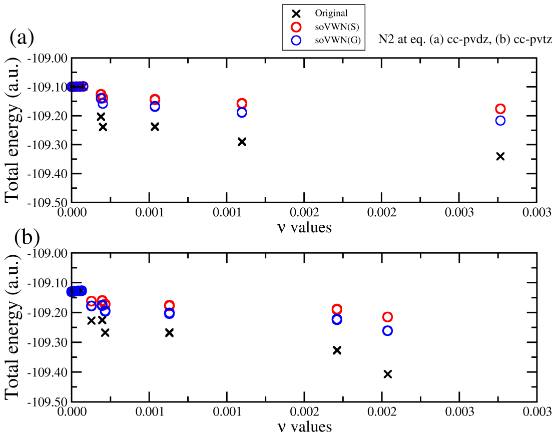

Figure S19: Total energy dependence on the parameter *nu* for the N_2 ground state at $r_{NN} = 1.10$ Å computed with the (a) cc-pVDZ and (b) cc-pVTZ basis sets.

5.4 Reducing discontinuities

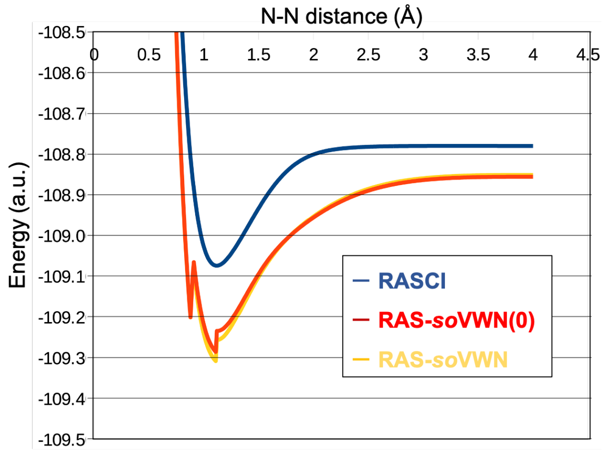

Figure S20: Absolute energy profiles along the N_2 dissociation computed at the RASCI (dark blue), RAS-*so*VWN (yellow), RAS-*so*VWN (orange) computed with the cc-pVDZ basis set. RAS-*so*VWN have been obtained by fixing the number of NOs entering the *so*VWN correlation to those corresponding whose sum of occupation numbers is lower than 0.001 at the equilibrium distance.

6 Size consistency analysis

Calculations for H_2 + nHe systems ($n = 1, 2, 3, 4$) with H$\cdots$He distances equal to 100 Å. RAS-*so*VWN(G) calculations with RAS2 space with 2 electrons in 2 orbitals, and threshold = 0.001 for the *so*DFT correlation. Reference: ROHF triplet.

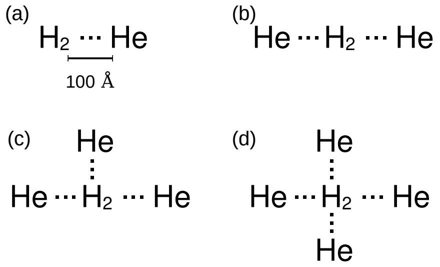

Figure S21: The different $H_2 \cdots n$He systems considered in this analysis. He atoms are placed orthogonally in the x- and y-axes, while H_2 bond lies on the z-axis.

Table S19: Energies of the total system, sum of fragment energies and absolute error computed for the ROHF triplet references, RAS-SF and RAS-*so*VWN(G) singlet ground state of the $H_2 \cdots$He system.

Basis set	ROHF	RAS-SF	*so*VWN(G)
Energy of total system (a.u.)			
cc-pVDZ	-3.6222	-4.0005	-4.0162
cc-pVTZ	-3.6355	-4.0111	-4.0278
cc-pVQZ	-3.6379	-4.0119	-4.0293
Sum of fragment energies (a.u.)			
cc-pVDZ	-3.6222	-4.0005	-4.0016
cc-pVTZ	-3.6355	-4.0111	-4.0120
cc-pVQZ	-3.6379	-4.0119	-4.0129
Absolute error (kcal/mol)			
cc-pVDZ	0.00	0.00	9.15
cc-pVTZ	0.00	0.00	9.91
cc-pVQZ	0.00	0.00	10.27

Table S20: Energies of the total system, sum of fragment energies and absolute error computed for the ROHF triplet references, RAS-SF and RAS-*so*VWN(G) singlet ground state of the $H_2\cdots 2He$ system.

Basis set	ROHF	RAS-SF	*so*VWN(G)
Energy of total system (a.u.)			
cc-pVDZ	-6.4774	-6.8557	-6.8763
cc-pVTZ	-6.4967	-6.8722	-6.8944
cc-pVQZ	-6.4994	-6.8734	-6.8964
Sum of fragment energies (a.u.)			
cc-pVDZ	-6.4774	-6.8556	-6.8568
cc-pVTZ	-6.4967	-6.8722	-6.8732
cc-pVQZ	-6.4994	-6.8734	-6.8744
Absolute error (kcal/mol)			
cc-pVDZ	0.00	0.04	12.25
cc-pVTZ	0.00	0.02	13.30
cc-pVQZ	0.01	0.03	13.77

Table S21: Energies of the total system, sum of fragment energies and absolute error computed for the ROHF triplet references, RAS-SF and RAS-*so*VWN(G) singlet ground state of the $H_2\cdots 3He$ system.

Basis set	ROHF	RAS-SF	*so*VWN(G)
Energy of total system (a.u.)			
cc-pVDZ	-9.3326	-9.7109	-9.7358
cc-pVTZ	-9.3578	-9.7334	-9.7602
cc-pVQZ	-9.3610	-9.7349	-9.7627
Sum of fragment energies (a.u.)			
cc-pVDZ	-9.3326	-9.7108	-9.7119
cc-pVTZ	-9.3578	-9.7334	-9.7343
cc-pVQZ	-9.3609	-9.7349	-9.7359
Absolute error (kcal/mol)			
cc-pVDZ	0.00	0.04	14.97
cc-pVTZ	0.01	0.02	16.24
cc-pVQZ	0.01	0.03	16.83

Table S22: Energies of the total system, sum of fragment energies and absolute error computed for the ROHF triplet references, RAS-SF and RAS-*so*VWN(G) singlet ground state of the $H_2 \cdots 4\,He$ system.

Basis set	ROHF	RAS-SF	*so*VWN(G)
Energy of total system (a.u.)			
cc-pVDZ	-12.1877	-12.5660	-12.5948
cc-pVTZ	-12.2190	-12.5945	-12.6255
cc-pVQZ	-12.2225	-12.5965	-12.6286
Sum of fragment energies (a.u.)			
cc-pVDZ	-12.1877	-12.5660	-12.5671
cc-pVTZ	-12.2190	-12.5945	-12.5955
cc-pVQZ	-12.2225	-12.5964	-12.5974
Absolute error (kcal/mol)			
cc-pVDZ	0.00	0.04	17.39
cc-pVTZ	0.01	0.02	18.87
cc-pVQZ	0.01	0.03	19.57

References

(S1) Casanova, D. Avoided crossings, conical intersections, and low-lying excited states with a single reference method: The restricted active space spin-flip configuration interaction approach. *J. Chem. Phys.* **2012**, *137*, 084105.

(S2) Wigner, E. On the interaction of electrons in metals. *Phys. Rev.* **1934**, *46*, 1002.

(S3) Löwdin, P.-O. Quantum theory of many-particle systems. I. Physical interpretations by means of density matrices, natural spin-orbitals, and convergence problems in the method of configurational interaction. *Phys. Rev.* **1955**, *97*, 1474.

(S4) Chakravorty, S. J.; Gwaltney, S. R.; Davidson, E. R.; Parpia, F. A.; p Fischer, C. F. Ground-state correlation energies for atomic ions with 3 to 18 electrons. *Phys. Rev. A* **1993**, *47*, 3649.

(S5) Huber, K. P.; Herzberg, G. *Molecular Spectra and Molecular Structure: IV. Constants of Diatomic Molecules*; Springer US: Boston, MA, 1979; pp 8–689.
